# Maturation and Protection Effect of Retinal Tissue-Derived Bioink for 3D Cell Printing Technology

**DOI:** 10.3390/pharmaceutics13070934

**Published:** 2021-06-23

**Authors:** Jongmin Kim, Jeong Sik Kong, Hyeonji Kim, Wonil Han, Jae Yon Won, Dong-Woo Cho

**Affiliations:** 1 Department of Mechanical Engineering, Pohang University of Science and Technology (POSTECH), Pohang 37673, Korea; mandarinbear@postech.ac.kr (J.K.); ghj901017@postech.ac.kr (H.K.); 2 School of Interdisciplinary Bioscience and Bioengineering, Pohang University of Science and Technology (POSTECH), Pohang 37673, Korea; urhere@postech.ac.kr; 3 Division of Integrative Biosciences and Biotechnology, Pohang University of Science and Technology (POSTECH), Pohang 37673, Korea; wihan@postech.ac.kr; 4 Department of Ophthalmology and Visual Science, Eunpyeong St. Mary’s Hospital, The Catholic University of Korea, Seoul 03312, Korea; 5 Catholic Institute for Visual Science, College of Medicine, The Catholic University of Korea, Seoul 14662, Korea; 6 Institute of Convergence Science, Yonsei University, Seoul 03722, Korea

**Keywords:** 3D cell printing, bioink, retina, tissue engineering, vision

## Abstract

Retinal degeneration is a leading cause of incurable vision loss and blindness. The increasing incidence of retinal degeneration has triggered research into the development of in vitro retinal models for drug development and retinal alternatives for transplantation. However, the complex retinal structure and the retinal microenvironment pose serious challenges. Although 3D cell printing technology has been widely used in tissue engineering, including in vitro model development and regeneration medicine, currently available bioinks are insufficient to recapitulate the complex extracellular matrix environment of the retina. Therefore, in this study, we developed a retinal decellularized extracellular matrix (RdECM) from the porcine retina and evaluated its characteristics. The RdECM conserved the ECM components from the native retina without cellular components. Then, we mixed the RdECM with collagen to form a bioink and confirmed its suitability for 3D cell printing. We further studied the effect of the RdECM bioink on the differentiation of Muller cells. The retinal protective effect of the RdECM bioink was confirmed through a retinal degeneration animal model. Thus, we believe that the RdECM bioink is a promising candidate for retinal tissue engineering.

## 1. Introduction

The retina is the light-sensitive layer of the ocular system and is composed of various cells that allow translation of the focused image to electrical signals, which are transferred to the brain for visual image processing [1,2]. Due to its crucial role in vision, damage to this layer breaks down the visual system and causes a wide range of impairments related to vision loss, such as age-related macular degeneration, diabetic retinopathy, and retinitis pigmentosa [3,4,5]. These impairments are the leading causes of global blindness, but no effective treatment exists. Extensive research has been conducted to develop retinal alternatives for retinal regeneration or to develop in vitro retina models for evaluation by pharmaceutical industries.

Transplantation is one of the promising treatments to replenish the damaged retina, for which several strategies have been adopted, including cell injection and the implantation of cell sheets [6,7,8,9]. It is essential to develop functionally mature retinal cells to treat patients with retinal dysfunction. Recently, human retinal progenitor cells were transplanted into patients with retinitis pigmentosa [10]. However, the cells used for transplantation were isolated from the ocular tissue of aborted fetuses through a labor-intensive process.

Various molecules, including small molecules, are reported to cure the damaged retina [11,12]. For this purpose, cells are cultured on a membrane with a small pore size, which allows the flow of the culture medium between the apical and the basal sides to evaluate drug performance [13]. This porous membrane-based system showed enhanced functionality compared with the traditional 2D culture system [14,15]. Recently, retinal organoid (RO) was reported as a model of the retina for drug development [16,17]. The RO with an organized retinal structure and various types of cells, including photoreceptors and ganglion cells, showed the reproduction of the adverse effect of antimalarial drugs [18]. However, the development of organoids is a time-consuming, laborious, and complex process.

A retinal construct with natural functions is essential for both transplantation and drug development. However, the traditional method does not allow fabrication of the retinal alternatives or establishing an in vitro model with controlled positions of multiple cell types of the retina. In addition, the retina is composed of various kinds of extracellular matrix (ECM) components [19]. Several materials, such as fibronectin, were used to mimic the retinal microenvironment; however, the complex retinal ECM environment is challenging to recapitulate [10,20].

Recently, 3D cell printing technology has been widely used to fabricate printed cell constructs for use in regeneration medicine and to establish in vitro models due to its ability to precisely deposit biomaterials and living cells according to their native equivalents [21,22,23]. Furthermore, advancements in bioink technology have also contributed to the recapitulation of the natural environment of the tissues [24,25,26].

In this study, we developed a retinal decellularized ECM (RdECM) bioink for 3D cell printing. The developed RdECM bioink was characterized by biochemical and rheological assays. Then, its printability and cytotoxicity were analyzed. Furthermore, its ability to induce the differentiation of Muller cells was compared with that of conventional collagen bioink. Finally, the protective effect of the RdECM in retinal degeneration was investigated through animal models. The results of this study suggest that the RdECM bioink could be used for the development of retinal alternatives and for establishing in vitro models.

## 2. Materials and Methods

### 2.1. Preparation of RdECM Bioink

The RdECM was developed through multiple processes. Retinal tissue was isolated from porcine eyes that were procured from the local slaughterhouse within 2 h. Briefly, the eyes were cleaned to remove surrounding tissues. The cleaned eyes were submerged in PBS and the cornea and the vitreous body were removed. Then, the retina was peeled off carefully by using a disposable pipette. The collected retina was washed with PBS overnight to remove blood in the retinal capillary. The washed retina was treated with 0.1% SDS in PBS for 6 h with gentle shaking. After washing with PBS three times, the retina was placed in 2% Triton-X/25 mM EDTA in PBS for 72 h. Then, the retina was washed with PBS six times and incubated with 100 U/mL DNase for 6 h. After PBS washing six times, the retina was sterilized with 0.1% peracetic acid in 4% ethanol for 2 h. Then, the sterilized retina was washed with PBS three times and then with distilled water. The developed RdECM was lyophilized and stored at −20 °C. For the preparation of bioink, the lyophilized RdECM was digested via pepsin–acetic acid under constant stirring. Finally, the digested RdECM was mixed with collagen (Dalim Tisen, Seoul, Korea) solution and neutralized using 0.1 N NaOH and 10× DMEM to a final concentration of 1% RdECM/1% collagen. For further studies, this RdECM/collagen mixture was referred to as the RdECM bioink.

### 2.2. Biochemical Characterization

To evaluate the cell removal and preservation of ECM components in the RdECM, biochemical assays were conducted. For the assessment of DNA, glycosaminoglycans (GAGs), collagen, lyophilized native tissues, and the RdECM were digested using papain (125 μg/mL) in 0.1 M sodium phosphate with 5 mM Na2-EDTA and 5 mM cysteine-HCL at pH 6.5 for 17 h at 60 °C. As a diluent, papain solution without tissue samples was used. The DNA content was quantified using a DNA quantitation kit (DNAQF, Sigma), according to the manufacturer’s protocol. The fluorescence intensity was measured using a fluorescence spectrophotometer (Gemini XPS Microplate Reader, Molecular Device; excitation wavelength: 350 nm, emission wavelength: 460 nm). Calf thymus DNA (Sigma-Aldrich, St. Louis, MO, USA) was used to generate a standard curve for DNA. In the analysis of GAGs, the tissue-digested solution had been stained with 1,9-dimethyl methylene blue, and the absorbance at 525 nm was read. The standard curve used for estimating the number of sulfated GAGs in the samples was plotted using chondroitin sulfate A (Sigma-Aldrich). Collagen content was determined using a total collagen assay kit (Biovision, Milpitas, CA, USA). The samples were prepared according to the manufacturer’s instructions, following measurement of the absorbance at 540 nm; hydroxyproline was used to prepare the standard curve.

### 2.3. Immunohistochemistry

The native and decellularized retinal tissues were fixed with 4% paraformaldehyde (PFA) overnight, washed with ultrapure water, dehydrated in graded alcohol, and embedded in paraffin. The embedded block was sectioned at 5 µm thickness and deparaffinized and rehydrated. Finally, the sections were stained with H&E (Abcam, Cambridge, MA, USA), Alcian blue (Abcam), and Masson’s trichrome (Sigma-Aldrich) according to the manufacturer’s instructions. The sections were imaged using Pannoramic Scan II (3DHISTECH, Budapest, Hungary).

### 2.4. Rheological Characterization

Rheological properties of the RdECM bioink were analyzed using the Discovery HR-1 rheometer system (TA instruments, New Castle, DE, USA) and compared with 1% collagen. The steady-state shear sweep method was used to measure viscosity, and the dynamic frequency sweep method was used to determine frequency-dependent storage (G′) and loss (G″) moduli.

### 2.5. Proteomic Analysis

The RdECM was lysed using RIPA buffer (Biosesang, Seongnam, Korea) supplemented with a proteinase inhibitor cocktail (Sigma), and the protein content was measured using a BCA protein assay kit (Pierce, Waltham, MA, USA). Then, the samples were treated with 8 M urea and 50 mM ammonium bicarbonate buffer for 3 h, 1 M dithiothreitol treatment for 2 h, 550 mM iodoacetamide for 1 h, and an additional 1 M dithiothreitol treatment for 30 min. Next, the samples were incubated with trypsin at 37 °C for 18 h, followed by desalting with Sep-Pak. Mass spectrometric analysis was performed using a Thermo Scientific Q Exactive Hybrid Quadrupole-Orbitrap instrument (Thermo Fisher Scientific, Waltham, MA, USA). According to the manufacturer’s instruction. The mass data were acquired automatically using Proteome Discoverer 2.4 (Thermo Scientific, USA) against the porcine protein UniProt database. The acquired data were categorized based on the PANTHER gene ontology (GO) classification system. The collected data were also annotated with a human Matrisome database (MatrisomeDB, http://matrisomeproject.mit.edu/).

### 2.6. Cell Culture

The human Muller cell line MIO-M1 (passage 26–29, Moorfields/Institute of Ophthalmology) and human bone marrow-derived mesenchymal stem cell (passage 4–6, Cefobio) were maintained in DMEM high glucose (Gibco) supplemented with 10% fetal bovine serum (Gibco) and penicillin–streptomycin (Gibco) at 37 °C in a 5% CO_2_ humidified incubator.

### 2.7. Printability Test

The RdECM bioink was printed using a 3DXP multi-head cell printer (TnR Biofab) under various conditions of nozzle diameter, feed rate, and pressure to optimize the printing conditions. Briefly, the syringe filled with RdECM bioink was loaded in the printing head, maintained at 4 °C by a Peltier unit. Then, printing was conducted under a preset feed rate and pressure followed by incubation at 37 °C for 30 min for crosslinking. The printed RdECM bioink was observed and imaged under a microscope (Zeiss, Oberkochen, Germany), and the images were analyzed using ImageJ software (Version 1.53c, 2020, National Institutes of Health, Bethesda, MD, USA).

### 2.8. Encapsulation of the Cell and Printing

At 80% confluence, the cells were dissociated with TrypLE (Gibco) and encapsulated in collagen or RdECM bioink at a concentration of 5 × 10^6^ cells/mL. The cell–encapsulated RdECM bioink or collagen was printed and incubated at 37 °C for gelation. Then, the samples were cultured in a culture medium.

### 2.9. Proliferation and Live/Dead Assay

The Cell Counting Kit-8 (CCK-8, Dojindo, Waltham, MA, USA) was used to measure the proliferation rate of the printed MIO-M1 cells. RdECM bioink or collagen containing 5 × 10^6^ cells/mL MIO-M1 was printed on a 48-well plate, and the cells were washed with PBS and incubated with CCK-8 solution for 1 h on days 1, 4, and 7. Then, the absorbance of the CCK-8 solution was measured using a microplate spectrophotometer (Molecular Devices). The Live/Dead assay kit (Thermo Fisher) was used to examine the viability of the MIO-M1 cells. Briefly, a printed bioink containing MIO-M1 cells was incubated with a culture medium containing calcein AM and ethidium homodimer-1 for 10 min. Then, the MIO-M1 cells were washed with PBS, and the fluorescence signal was observed under a fluorescence microscope (Zeiss).

### 2.10. Immunostaining

The printed bioink containing the MIO-M1 cells was fixed with 4% PFA for 30 min at room temperature, followed by permeabilization with 0.1% Triton-X for 7 min. The samples were blocked with 10% normal goat serum (Vector Laboratory, San Francisco, CA, USA) for 1 h and incubated with a 1:100 dilution of blue opsin (Millipore, Burlington, MA, USA), red-green opsin (Millipore), rhodopsin (Abcam), glial fibrillary acidic protein (GFAP; Abcam), and glutamine synthetase (GS, Abcam) antibodies overnight at 4 °C (Table S1). After washing with PBS containing Tween-20 (PBST) three times, the samples were incubated with a 1:1000 dilution of secondary antibody conjugated with Alexa Fluor 488 or 594 (Invitrogen, Carlsbad, CA, USA). Then, the samples were washed with PBST three times and co-stained with DAPI for visualizing the nuclei. The fluorescence signal was observed under a fluorescence microscope (Zeiss).

### 2.11. Ethics Statement

All animal experiments were performed in strict accordance with a protocol approved by the Ethics Committee for Animal Experiments of St. Mary’s Hospital, the Catholic University of Korea (IACUC: EPSMH2020170301FA). All surgical operations were performed under anesthesia with 2% inhaled isoflurane via tracheal intubation with an 18-gauge intravenous catheter. All efforts were made to minimize animal suffering.

### 2.12. Animal Care

All mice used in this study were maintained in the specific pathogen-free area at St. Mary’s Hospital, the Catholic University of Korea. The mice were housed in a temperature-controlled room with a 12/12 h light/dark cycle and ad libitum access to food and water. All mice were monitored daily.

### 2.13. Animal Experiments

To confirm the protective effect of the RdECM, it was injected in a LASER-induced choroidal neovascularization (Laser-CNV) or a *N*-methyl-*N*-nitroso urea (NMU)-treated retinal degeneration (NMU-RD) model of C57BL6 (8 weeks, male, Orientbio) mice according to a reported procedure [27,28]. The Laser-CNV model (*n* = 5) was established by anesthetizing mice with inhaled isoflurane and topical proparacaine. Then, four points surrounding the optic nerve were shot with green LASER (wavelength: 532 nm; spot size: 50 µm, power: 100 mW, and exposure time: 0.1 s). Two weeks after LASER treatment, 2 µL Avastin or 0.1% RdECM or collagen were intravitreally injected into each group, respectively. After 1 week, FITC-dextran (molecular weight: 2 × 10^6^, 50 mg/mL, Sigma) was used for vascular perfusion according to a previously published report [29]. The mice were sacrificed and their eyes were collected and fixed with 4% PFA. The randomly selected lesion was chosen, and the size of the LASER irradiated lesion and vascular structure was observed under a fluorescence microscope (Zeiss) according to the previous report [30,31]. The remaining vascular area was calculated via the ratio of the size of the vascular structure and the LASER irradiated lesion. To establish the NMU-RD model, mice (*n* = 5) were intraperitoneally injected with 60 mg/kg NMU. After 2 days, 2 µL RdECM or collagen was intravitreally injected. After 1 week, the mice were sacrificed, and the retinal tissues were isolated, sectioned for histological analysis, and stained with H&E. The sections were then imaged using a Pannoramic Scan II (3DHISTECH). The thickness of the retina was measured using ImageJ software.

### 2.14. Statistical Analysis

The results were analyzed with GraphPad 8 (GraphPad Software, San Diego, CA, USA). The Student’s *t*-test and two-way analysis of variance (ANOVA) were applied to mean comparisons.

## 3. Results

### 3.1. Biochemical Characterization

**Figure 1:**
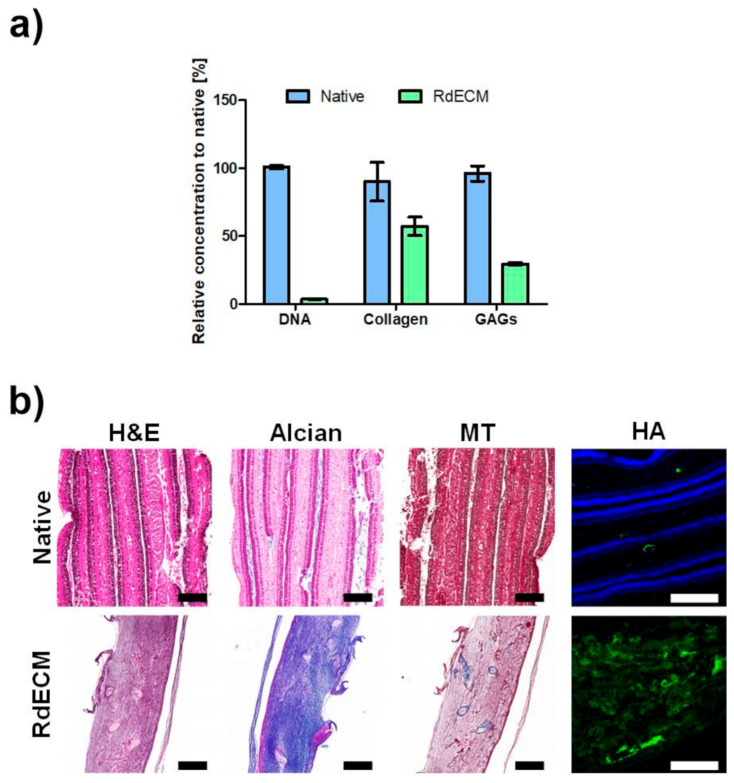
Characterization of retinal decellularized extracellular matrix (RdECM). (**a**) Biochemical analysis of collagen and glycosaminoglycans (GAGs) in the native retina (Native) and RdECM. (**b**) Immunohistochemical analysis of native retina (Native) and RdECM. Scale bar: 200 µm. The error bars represent the standard deviation.

The RdECM bioink was developed from the porcine retina through multiple processes including chemical and enzymatic treatment, lyophilization, and pepsin digestion (Figure S1). The ECM components present in the RdECM were analyzed through biochemical assay. Comparing with native retina tissue, 57.143 ± 6.734% of collagen and 29.592 ± 0.835% of GAGs were preserved, whereas only 3.567 ± 0.149% of DNA content remained (Figure 1a). Immunohistochemistry results also confirmed the conservation of collagen and GAGs, the elimination of nuclear components, and that hyaluronic acid was well preserved (Figure 1b).

### 3.2. Proteomic Analysis

**Figure 2:**
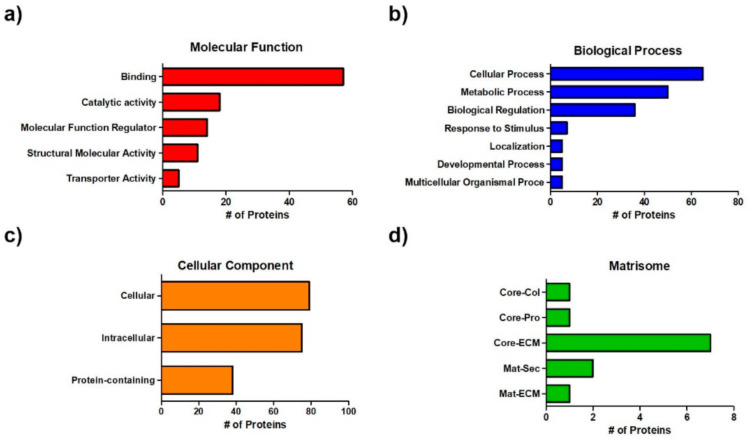
Proteomic analysis of retinal decellularized extracellular matrix (RdECM). (**a**) Molecular function. (**b**) Biological process. (**c**) Cellular component. (**d**) Matrisome (Core-Col: Core matrisome-Collagens, Core-Pro: Core matrisome-Proteoglycans, Core-ECM: Core matrisome-ECM Glycoproteins, Mat-Sec: Matrisome-associated secreted factors, Mat-ECM: Matrisome-associated ECM-affiliated proteins).

The components of the RdECM were identified through mass spectrometric analysis and subjected to PANTHER classification. The RdECM was found to contain various protein components related to the classification of molecular function, biological process, and cellular component (Figure 2a–c and Table S2). It also contained matrisomes, which are secreted proteins related to the extracellular matrix, and matrisome-associated proteins. RdECM contained “core matrisomes,” composed of structural protein components, such as collagens, glycoproteins, and proteoglycans, and “matrisome-associated proteins”, including ECM-affiliated proteins and secreted factors (Figure 2c and Table S3).

### 3.3. Rheological Characterization

**Figure 3:**
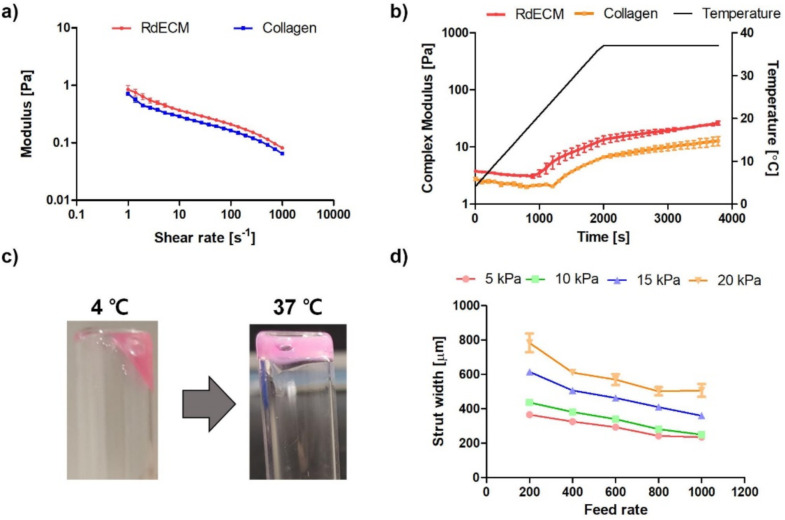
Rheological analysis and printability test of retinal decellularized extracellular matrix (RdECM). (**a**) Viscosity. (**b**) Complex modulus. (**c**) Sol-gel transition test. (**d**) Printability test (30-gauge nozzle). The error bars represent the standard deviation.

The rheological characteristics of the developed RdECM bioink were analyzed based on the viscosity and the dynamic modulus. The RdECM bioink showed decreasing viscosity as the shear rate increased (Figure 3a). The storage modulus was higher than the loss modulus (Figure S2). The complex modulus rapidly increased as the temperature increased from 20 °C to 30 °C (Figure 3b). The sol-gel transition was observed, indicating thermosensitive crosslinking. The RdECM bioink solution changed to the gel state at 37 °C (Figure 3c).

### 3.4. Printability Test

The printability of the bioink was studied under various nozzle diameters, feed rates, and pressures (Figure 3d and Figure S3). The width of the strut decreased as the feed rate increased. Furthermore, the strut width increased as the pressure or nozzle diameter increased.

### 3.5. Proliferation and Viability of the Printed Structure

**Figure 4:**
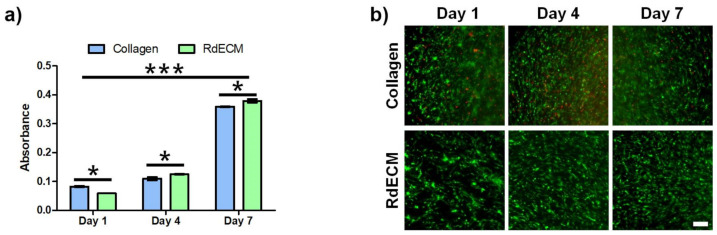
Proliferation and viability of printed MIO-M1 cells. (**a**) Proliferation and (**b**) Live/Dead assay of MIO-M1 cells encapsulated in retinal decellularized extracellular matrix (RdECM) bioink or collagen. The error bars represent the standard deviation. Scale bar: 100 µm. The data were compared using two-way ANOVA and differences were considered significant when * for *p* < 0.05 and *** for *p* < 0.001.

The proliferation and viability of the printed MIO-M1 cells encapsulated in the RdECM bioink were compared with those of the cells encapsulated in collagen (Figure 4a,b). The proliferation of the MIO-M1 cells in collagen was higher than that of the cells in the RdECM bioink on day 1; however, higher proliferation was observed in the RdECM bioink during days 4 to 7. Live cells were observed in the RdECM bioink during the culture period, whereas dead cells were observed in the collagen.

### 3.6. Effect of RdECM in Muller Cell Differentiation

**Figure 5:**
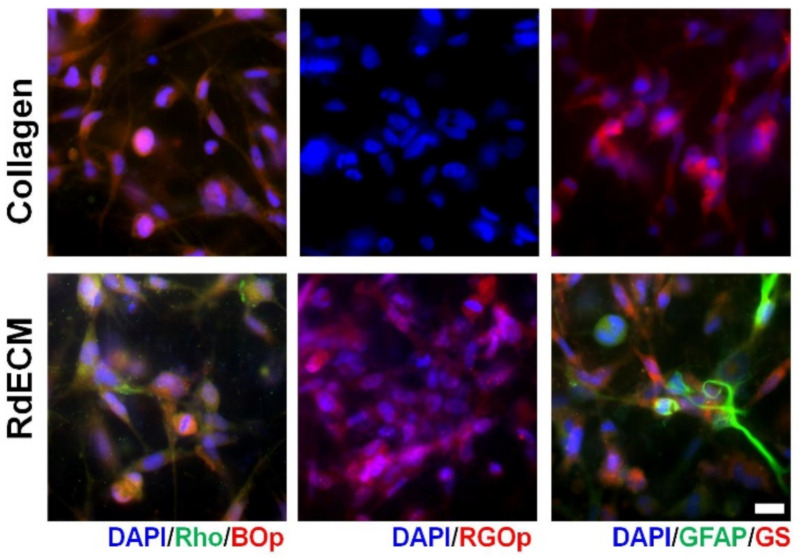
Effect of retinal decellularized extracellular matrix (RdECM) bioink for Muller cell differentiation (rhodopsin: Rho, blue opsin: BOp, red-green opsin: RGOp, glial fibrillary acidic protein: GFAP, glutamine synthetase: GS). Scale bar: 20 µm.

The expression of retinal markers was compared between the MIO-M1 cells encapsulated in the RdECM bioink and those in the collagen (Figure 5). The expression of photoreceptor markers, including blue opsin, red-green opsin, and rhodopsin was observed in the RdECM bioink-encapsulated cells, whereas only blue opsin was expressed in the collagen samples. The glial marker glutamine synthetase was observed in both bioinks; however, GFAP was only expressed in the RdECM bioink.

### 3.7. Protective Effect of RdECM in Laser-CNV and NMU-RD model

**Figure 6:**
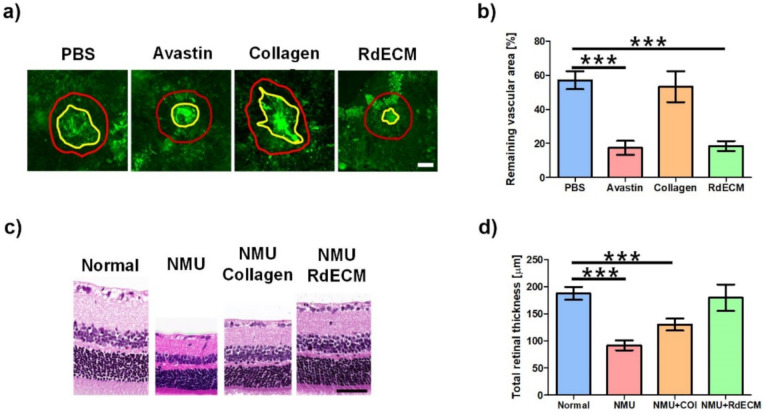
Retinal protective effect of retinal decellularized extracellular matrix (RdECM). (**a**) FITC-dextran image of the retina from Laser-CNV model mice. Red: LASER irradiated lesion. Yellow: vascular structure. (**b**) Remaining vascular area in the Laser-CNV model. (**c**) H&E staining images of the retina from NMU-RD model mice. (**d**) Total retinal thickness in the NMU-RD model. The error bars represent the standard deviation. The data were compared using the Student’s t-test and differences were considered significant when *** for *p* < 0.001. Scale bar: (**a**) 100 µm, (**c**) 50 µm.

The retinal protective effect of the RdECM was confirmed in the Laser-CNV and the NMU-RD models (Figure 6a–d). The size of the wound in the CNV model decreased in the RdECM-treated mice and showed a similar size with that of the Avastin-treated mice, whereas it was nearly unchanged in the collagen-treated mice. In the NMU-RD model, retinal thinning was observed when compared with the normal mice (50% of normal retinal thickness). Both the collagen and the RdECM showed a retinal protective effect. The RdECM-treated NMU-RD model mice showed a 95% normal retinal thickness, whereas this was 69% in the collagen-treated mice.

## 4. Discussion

The retina is composed of various kinds of cells and plays a crucial role in the human visual system. Cells in the retina convert visual information to electrical signals, which are transported to the visual areas of the brain. Disruption of this circuit causes vision loss, including age-related macular degeneration and retinitis pigmentosa. Currently, there is no effective cure for these diseases. Recently, retinal progenitor cells (RPCs) have been used as a transplantation strategy for treating retinitis pigmentosa [32,33]. Despite injecting the fetal RPCs, their visual acuity was nearly unchanged [10].

Previous studies showed that the injection of the cells is insufficient to treat retinal degeneration since the retina has a complex structure and ECM environment. To overcome this limitation, scaffold-based implantation was conducted [34,35]. Singh et al. developed a porous scaffold using gelatin, chondroitin sulfate, and hyaluronic acid to culture embryonic stem cell–derived retinal progenitor cells [36]. Redenti et al. used polycaprolactone to fabricate a scaffold with mouse RPCs as nanowires with an optimized length [37]. Similarly, an intraocular lens injector system was used to prepare a flexible polyglycerol sebacate membrane [38]. Various strategies were reported for preparing the retinal scaffold; however, the materials used, and the conventional 2D culture method, could not recapitulate the complex retinal microenvironment.

Recently, 3D printing and tissue-specific bioink have been widely used in tissue engineering, including regeneration medicine and drug-testing models [21]. The advanced 3D printing system could simultaneously print various materials, including synthetic and natural polymers. In addition, organ-derived bioink could provide optimized ECM components to the target organs [39,40,41]. The integration of these two features facilitates the development of a tissue analog with a 3D biomimetic structure.

For the adoption of 3D cell printing in the ocular system, we aimed to develop a retina-derived bioink. We isolated retinal tissue from porcine eyes and optimized the decellularization process, including chemical and enzyme treatment. The developed RdECM preserved ECM components such as collagen and GAGs, whereas the cellular components were removed. For further analysis, we conducted mass spectrometry. In terms of the cellular component, the cell part (GO:0044464) and organelle part (GO:0005622) were removed, which indicated that the decellularization process was optimized. The RdECM also contained components of the biological process related to tissue/organ modeling, such as the developmental process (GO:0032502) and biological adhesion (GO:0022610). In addition, the components for molecular function, such as binding (GO:0005488) and structural molecule activity (GO:0005198) were observed. Furthermore, the RdECM contained proteins related to ECM called matrisomes [42]. Matrisomes are composed of core structural proteins called core matrisome proteins (collagens, glycoproteins, and proteoglycans) and matrisome-associated proteins (ECM regulators, ECM-affiliated proteins, and secreted factors), which play a crucial role in cell function [43,44]. Both components were identified in the RdECM, especially tenascin-R, related to the central nervous system, and laminin beta-2, which is the key component for providing environmental cues to the RPCs [45,46]. These results indicated that the developed RdECM contained components related to various cellular functions.

Then, the bioink composition was optimized based on cell proliferation. We mixed the RdECM with conventional collagen bioink to obtain suitable printing characteristics. Comparing with 1% collagen bioink, the cells proliferated well in the composition of 1% RdECM/1% collagen, while the lower proliferation was observed in a higher RdECM concentration (Figure S4). Therefore, we chose the 1% RdECM/1% collagen composition as the RdECM bioink for further study.

The rheological characteristic and printability were analyzed for 3D bioprinting. The developed RdECM bioink showed decreased viscosity at higher shear rates, indicating a shear-thinning behavior, which is one of the important properties of a bioink because it can protect cells from shear stress and prevent clogging during the printing process [47,48]. The stability of the crosslinked RdECM bioink was evaluated by measuring the dynamic modulus. The storage modulus of the RdECM bioink was higher than the loss modulus, indicating that the RdECM bioink was transformed from a solution to a gel-like structure. Notably, a larger modulus was measured in the RdECM bioink than in the collagen bioink. These results indicated that the complex ECM components of the RdECM affect the structure of the bioink and enhance substrate stability under dynamic conditions. Furthermore, the RdECM was tested for printability under various printing conditions. The width of the strut of the printed RdECM bioink decreased as the feed rate increased and pressure and nozzle size decreased. However, the printing failed when using a 25-gauge nozzle at a high feed rate and using a 27-gauge nozzle at high pressure. Therefore, the 30-gauge nozzle was chosen due to its versatility in feed rate and pressure conditions.

The proliferation and viability of the printed MIO-M1 cells were analyzed via CCK-8 and Live/Dead cell assays. The cells proliferated well in both bioinks; however, a higher proliferation rate was observed for the MIO-M1 cells encapsulated in the RdECM bioink. In addition, most of the cells proliferated well in the RdECM bioink during the culture period, whereas damaged cells were observed in the collagen samples. These results indicated that the developed RdECM bioink is suitable for 3D cell printing.

The ECM plays a crucial role in the functional maturation of the ocular system, including photoreceptor organization and ganglion cell differentiation [49]. Therefore, we compared the expression of retinal markers in the MIO-M1 cells encapsulated in the RdECM bioink and the collagen. The MIO-M1 cells in both bioinks showed photoreceptor and glial cell characteristics by expressing blue opsin and glutamine synthetase as reported [50]. However, the expression of red-green opsin, rhodopsin, and GFAP was observed only in the cells encapsulated in the RdECM bioink. Muller cells provide structural support and functional stability to the retina [51]. Recently, Muller cells have been suggested to be a potential candidate for treating retinal degeneration [52]. Giannelli et al. showed the differentiation ability of primary human Muller cells into photoreceptor cells using a feeder layer culture [53]. Lawrence et al. showed the expression of a retinal marker when cells were grafted into the subretinal layer of rats [54]. These studies and our results indicate that external conditions could induce the differentiation of Muller cells into retinal component cells, verifying that the RdECM bioink promotes the maturation of Muller cells.

Furthermore, we confirmed the retinal protective ability of the RdECM using the Laser-CNV and the NMU-RD model due to their wide use as retinal degeneration models, including AMD and RP [55,56,57,58]. The RdECM-treated Laser-CNV mice showed similar wound recovery as the Avastin-treated mice, whereas the recovery was nearly unchanged in the collagen-treated mice. A similar result was observed in the NUM-RD model mice. The retina in the RdECM-treated mice had similar thicknesses with untreated samples because of the preserved ECM components of the RdECM. The RdECM is composed of various kinds of ECM components derived from the native retina. The RdECM specifically contains hyaluronic acid, heparin sulfate, and laminin, which are the main ECM components of the retina [19,59]. Various studies reported that these kinds of ECM components could enhance the function and promote the maturation of retinal cells [49]. These characteristics might protect the retina against physical and chemical damage and might be one of the candidates for retinal repairment for AMD or RP.

## 5. Conclusions

The RdECM bioink was developed from the porcine retina via a decellularization process. The developed bioink contained various kinds of ECM components and was suitable for 3D cell printing. Compared with conventional the MIO-M1 cells in collagen, the MIO-M1 cells in the RdECM bioink showed a retinal differentiation tendency. Furthermore, the retina protection ability was also confirmed through the Laser-CNV and the NMU-RD models. Therefore, the RdECM is an appropriate candidate for developing a retinal alternative for use in regeneration medicine or as an in vitro drug-testing platform.

## Data Availability

Not applicable.

## Supplementary Materials

The following are available online at https://www.mdpi.com/article/10.3390/pharmaceutics13070934/s1, Figure S1: Development procedure of retinal decellularized extracellular matrix (RdECM), Figure S2: Storage and loss modulus of retinal decellularized extracellular matrix (RdECM) bioink and collagen. The error bars represent the standard deviation, Figure S3: Strut width of printed retinal decellularized extracellular matrix (RdECM) bioink using (a) 25-gauge nozzle and (b) 27-gauge nozzle. The error bars represent the standard deviation, Figure S4: Proliferation of mesenchymal stem cell in various compositions of RdECM bioink. C1: 1% Collagen. C1/R1: 1% collagen/1% RdECM. C1/R2: 1% collagen/2% RdECM. C1/R3: 1% collagen/3% RdECM, Table S1: List of antibodies, Table S2: Mass spectrometric of RdECM using Sus scrofa UniProt database search, Table S3: Matrisome compoenents of retinal decellularized extracellular matrix (RdECM).
