# Supplementary material for: Maturation and Protection Effect of Retinal Tissue-Derived Bioink for 3D Cell Printing Technology
Source: Pharmaceutics. 2021 Jun 23;13(7):934. doi: 10.3390/pharmaceutics13070934 (PMC8309106; doi:10.3390/pharmaceutics13070934)
Supplement: Supplementary file 1 [file pharmaceutics-13-00934-s001.zip › pharmaceutics-1252453-supplementary.pdf]

pharmaceutics

Supplementary Materials: Maturation and Protection Effect of Retinal Tissue-Derived Bioink for 3D Cell Printing Technology

Jongmin Kim, Jeong Sik Kong, Hyeonji Kim, Wonil Han, Jae Yon Won and Dong-Woo Cho

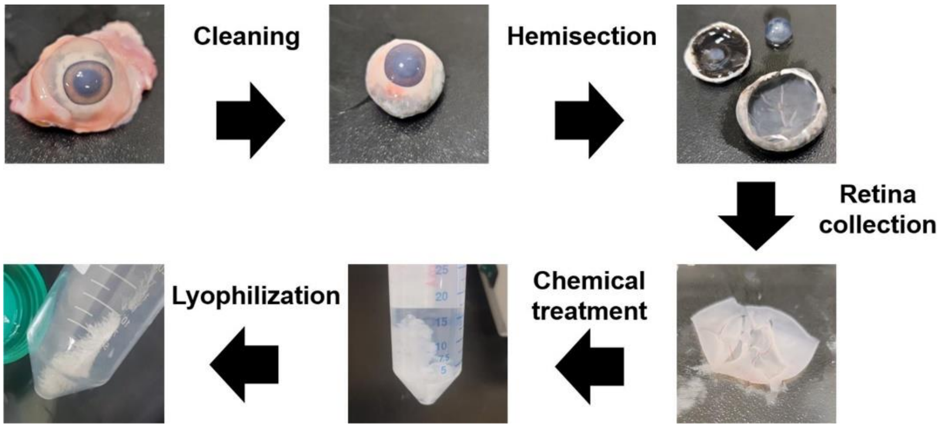

Figure S1. Development procedure of retinal decellularized extracellular matrix (RdECM).

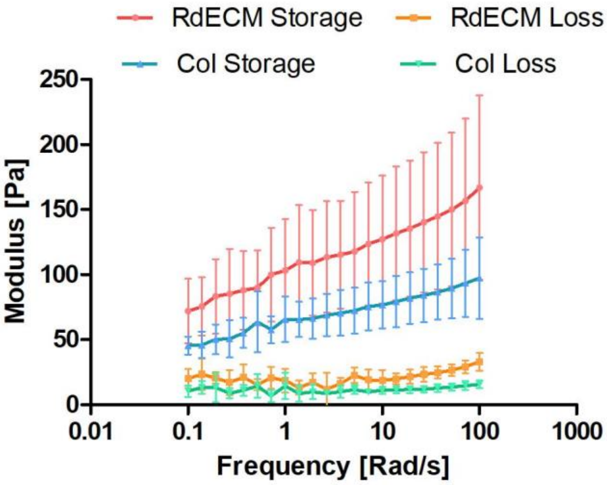

Figure S2. Storage and loss modulus of retinal decellularized extracellular matrix (RdECM) bioink and collagen. The error bars represent the standard deviation.

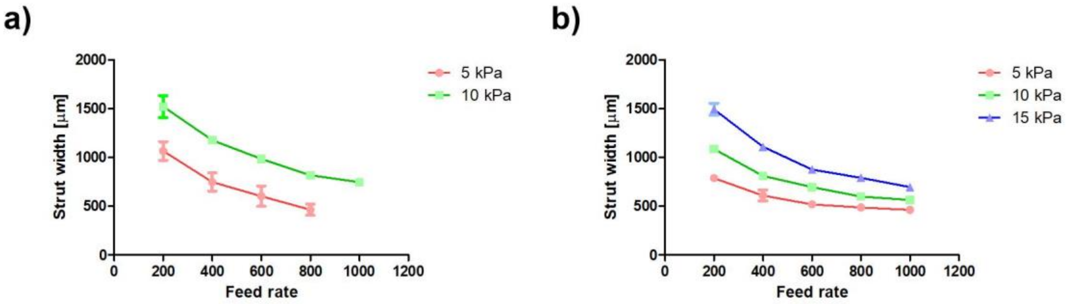

Figure S3. Strut width of printed retinal decellularized extracellular matrix (RdECM) bioink using **(a)** 25-gauge nozzle and **(b)** 27-gauge nozzle. The error bars represent the standard deviation.

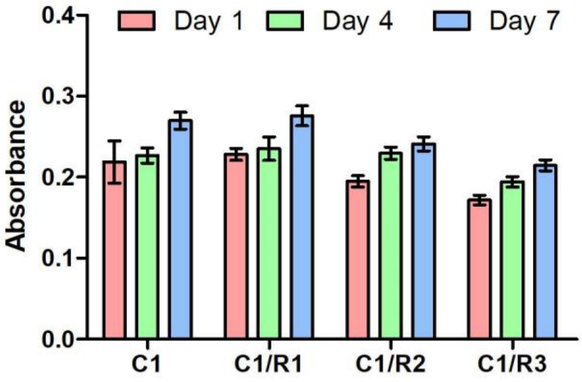

Figure S4. Proliferation of mesenchymal stem cell in various compositions of RdECM bioink. C1: 1% Collagen. C1/R1: 1% collagen/ 1% RdECM. C1/R2: 1% collagen/ 2% RdECM. C1/R3: 1% collagen/ 3% RdECM.

Table S1. List of antibodies.

Name	Manufacture	Host	Cat #
Blue opsin	Millipore	Rabbit	AB5407
Red-Green opsin	Millipore	Rabbit	AB5405
Rhodopsin	Abcam	Mouse	ab5417
GFAP	Abcam	Mouse	ab4648
GS	Abcam	Rabbit	ab176562

Table S2. Mass spectrometric of RdECM using Sus scrofa UniProt database search.

Checked	Master	Accession	Description	Coverage [%]	# Peptides	# PSMs	# Unique Peptides	# AAs	MW [kDa]	calc. pI	Score Sequest HT: Sequest HT	# Peptides (by Search Engine): Sequest HT	# Protein Groups
TRUE	Master Protein	A0A480M929	Tubulin beta chain OS=Sus scrofa OX=9823 PE=3 SV=1	73	25	93	2	445	49.9	4.89	320.88	25	1
TRUE	Master Protein	P02543	Vimentin OS=Sus scrofa OX=9823 GN=VIM PE=1 SV=2	65	34	99	30	466	53.6	5.12	318.74	34	1
TRUE	Master Protein	P62802	Histone H4 OS=Sus scrofa OX=9823 PE=1 SV=2	57	10	99	10	103	11.4	11.36	316.95	10	1
TRUE	Master Protein	A0A480VHK4	Tubulin beta chain OS=Sus scrofa OX=9823 PE=3 SV=1	72	24	91	0	445	49.8	4.89	309.82	24	1
TRUE	Master Protein	Q71LE2	Histone H3.3 OS=Sus scrofa OX=9823 GN=H3-3A PE=2 SV=3	60	7	74	2	136	15.3	11.27	307.17	7	1
TRUE	Master Protein	A0A287AZ37	Tubulin beta chain OS=Sus scrofa OX=9823 GN=LOC100624785 PE=1 SV=2	69	24	88	1	465	52	5.05	301.27	24	1
TRUE	Master Protein	F2Z5B2	Tubulin beta chain OS=Sus scrofa OX=9823 GN=TUBB PE=1 SV=2	70	23	87	3	459	51.2	7.28	285.47	23	1
TRUE	Master Protein	A0A287BFK0	Histone H2A OS=Sus scrofa OX=9823 GN=H2AW PE=3 SV=1	64	8	68	2	130	14.1	11.05	278.66	8	1
TRUE	Master Protein	A0A480UV25	Tubulin beta chain OS=Sus scrofa OX=9823 PE=3 SV=1	70	22	82	2	444	49.6	4.88	278.52	22	1
TRUE	Master Protein	A0A480PJ61	Histone H2A OS=Sus scrofa OX=9823 PE=3 SV=1	70	10	54	5	143	15.1	10.74	251.97	10	1
TRUE	Master Protein	A0A5G2QML3	Histone H2B OS=Sus scrofa OX=9823 GN=H1-3 PE=1 SV=1	41	19	81	4	322	33.5	10.74	245.57	19	1
TRUE	Master Protein	F2Z5L5	Histone H2A OS=Sus scrofa OX=9823 GN=H2AC20 PE=1 SV=1	64	8	59	2	129	14	10.9	236.21	8	1
TRUE	Master Protein	F1RJU4	Neurofilament light polypeptide OS=Sus scrofa OX=9823 GN=NEFL PE=1 SV=1	49	30	68	27	552	62.4	4.61	216.45	30	1
TRUE	Master Protein	A0A4X1WCU9	Tubulin alpha chain OS=Sus scrofa OX=9823 GN=TUBA1A PE=3 SV=1	60	16	60	1	416	46.3	5.08	207.3	16	1
TRUE	Master Protein	A0A0B8RZ36	Spectrin, alpha, non-erythrocytic 1 OS=Sus scrofa domesticus OX=9825 GN=SPTAN1 PE=3 SV=1	26	49	67	49	2452	281.9	5.3	204.87	49	1
TRUE	Master Protein	A0A480IZE0	Tubulin alpha chain OS=Sus scrofa OX=9823 PE=3 SV=1	49	15	56	0	448	49.9	5.06	196.06	15	1
TRUE	Master Protein	A0A480VY62	160 kDa neurofilament protein OS=Sus scrofa OX=9823 PE=3 SV=1	21	19	57	4	886	99.1	4.87	182.21	19	1
TRUE	Master Protein	A0A480NAY0	Tubulin alpha chain OS=Sus scrofa OX=9823 PE=3 SV=1	45	13	50	1	449	50.1	5.1	181.98	13	1
TRUE	Master Protein	A0A287B3D7	Core histone macro-H2A OS=Sus scrofa OX=9823 GN=MACROH2A1 PE=1 SV=2	57	14	44	12	369	39.2	9.83	180.13	14	1

TRUE	Master Protein	A0A480IV89	Tubulin alpha chain OS=Sus scrofa OX=9823 PE=3 SV=1	47	14	50	1	448	49.9	5.06	178.85	14	1
TRUE	Master Protein	P08552	Neurofilament medium polypeptide (Fragment) OS=Sus scrofa OX=9823 GN=NEFM PE=1 SV=2	38	16	54	1	455	52	5.24	173.46	16	1
TRUE	Master Protein	F1RVA0	Histone H3 OS=Sus scrofa OX=9823 GN=HIST1H3E PE=2 SV=2	52	6	40	1	136	15.4	11.12	165.16	6	1
TRUE	Master Protein	A0A287BID4	Histone H2B OS=Sus scrofa OX=9823 GN=H2BC21 PE=3 SV=1	62	10	53	2	126	13.9	10.32	164.3	10	1
TRUE	Master Protein	A0A286ZJQ0	Histone H3 OS=Sus scrofa OX=9823 PE=3 SV=1	52	6	39	1	136	15.4	11.27	162.29	6	1
TRUE	Master Protein	F1RR02	Glial fibrillary acidic protein OS=Sus scrofa OX=9823 GN=GFAP PE=1 SV=3	56	26	52	25	468	53.6	5.85	162.08	26	1
TRUE	Master Protein	Q6QAQ1	Actin, cytoplasmic 1 OS=Sus scrofa OX=9823 GN=ACTB PE=1 SV=2	62	15	46	1	375	41.7	5.48	157.14	15	1
TRUE	Master Protein	A0A4X1TEF4	Tubulin beta chain OS=Sus scrofa OX=9823 PE=3 SV=1	41	17	47	4	455	50.2	8.44	144.93	17	1
TRUE	Master Protein	A0A4X1W6K9	Spectrin beta chain OS=Sus scrofa OX=9823 PE=3 SV=1	21	34	42	33	2361	274.1	5.58	138.01	34	1
TRUE	Master Protein	F1S847	Alpha-internexin OS=Sus scrofa OX=9823 GN=INA PE=1 SV=1	48	22	43	19	499	55.4	5.4	135.42	22	1
TRUE	Master Protein	Q4TTS4	Histone H1.2-like protein OS=Sus scrofa OX=9823 PE=3 SV=1	39	13	38	4	213	21.4	11	109.75	13	1
TRUE	Master Protein	A0A4X1VU50	Tubulin beta chain OS=Sus scrofa OX=9823 GN=TUBB6 PE=3 SV=1	29	11	32	0	444	49.4	5.48	103.99	11	1
TRUE	Master Protein	A0A480UP67	Myosin-10 isoform 2 OS=Sus scrofa OX=9823 PE=3 SV=1	16	22	29	16	1976	228.7	5.55	100.57	22	1
TRUE	Master Protein	A0A4X1TCT0	Vesicle-fusing ATPase OS=Sus scrofa OX=9823 GN=NSF PE=3 SV=1	30	19	27	19	747	83	6.81	89.33	19	1
TRUE	Master Protein	A0A480QL86	Myosin-9 OS=Sus scrofa OX=9823 PE=3 SV=1	14	20	27	14	1967	226.7	5.53	88.45	20	1
TRUE	Master Protein	Q3ZD69	Prelamin-A/C OS=Sus scrofa OX=9823 GN=LMNA PE=3 SV=1	27	14	25	14	664	74.2	7.18	83.82	14	1
TRUE	Master Protein	A0A286ZVL0	Heterogeneous nuclear ribonucleoprotein U OS=Sus scrofa OX=9823 GN=HNRNPU PE=1 SV=1	19	14	28	14	825	90.6	5.86	83.08	14	1
TRUE	Master Protein	F1RFH3	Neurofilament heavy polypeptide OS=Sus scrofa OX=9823 GN=NEFH PE=1 SV=2	16	14	27	11	847	93.4	6.13	82.45	14	1
TRUE	Master Protein	A0A4X1V2S2	Peptidase S1 domain-containing protein OS=Sus scrofa OX=9823 GN=LOC100302368 PE=4 SV=1	24	4	21	4	246	25.9	7.18	75.85	4	1
TRUE	Master Protein	F1RKM0	Lamin-B1 isoform 1 OS=Sus scrofa OX=9823 GN=LMNB1 PE=1 SV=2	42	16	24	16	586	66.4	5.14	73.29	16	1
TRUE	Master Protein	A0A287AKA3	Core histone macro-H2A OS=Sus scrofa OX=9823 GN=MACROH2A2 PE=1 SV=1	48	11	18	9	372	40	9.69	69.77	11	1
TRUE	Master Protein	A0A4X1UCH7	Histone H2A OS=Sus scrofa OX=9823 GN=LOC100512448 PE=3 SV=1	43	4	19	2	128	13.5	10.58	66.19	4	1

TRUE	Master Protein	F1SU06	Heterochromatin protein 1-binding protein 3 OS=Sus scrofa OX=9823 GN=HP1BP3 PE=1 SV=2	29	15	22	15	553	61.1	9.72	66.05	15	1
TRUE	Master Protein	A0A480EWB1	Histone H1.1 OS=Sus scrofa OX=9823 PE=3 SV=1	29	8	22	1	218	22	10.87	64.6	8	1
TRUE	Master Protein	K7GND3	RNA helicase OS=Sus scrofa OX=9823 GN=DHX9 PE=1 SV=2	14	13	17	13	1384	149.6	6.98	63.94	13	1
TRUE	Master Protein	P06348	Histone H1t OS=Sus scrofa OX=9823 GN=H1-6 PE=1 SV=1	20	6	20	1	211	22	11.58	59.39	6	1
TRUE	Master Protein	A0A481CYB2	Actin, alpha skeletal muscle OS=Sus scrofa OX=9823 PE=3 SV=1	31	9	20	3	377	42	5.39	53.65	9	1
TRUE	Master Protein	A0A4X1V8T4	H15 domain-containing protein OS=Sus scrofa OX=9823 GN=LOC100510904 PE=3 SV=1	24	6	19	6	194	20.9	10.9	49.47	6	1
TRUE	Master Protein	A0A4X1U498	Heterogeneous nuclear ribonucleoproteins A2/B1 OS=Sus scrofa OX=9823 GN=HNRNPA2B1 PE=4 SV=1	41	10	16	10	361	38.4	9.07	46.65	10	1
TRUE	Master Protein	A0A4X1UY93	Heterogeneous nuclear ribonucleoprotein K OS=Sus scrofa OX=9823 GN=HNRNPK PE=4 SV=1	33	10	14	10	426	46.9	5.44	44.62	10	1
TRUE	Master Protein	A0A4X1UYH7	RRM domain-containing protein OS=Sus scrofa OX=9823 GN=HNRNPC PE=3 SV=1	25	7	14	7	317	34.9	5.08	42.47	7	1
TRUE	Master Protein	F1SP93	H(+)-transporting two-sector ATPase OS=Sus scrofa OX=9823 GN=ATP6V1A PE=1 SV=2	24	9	12	9	603	66.6	5.33	42.44	9	1
TRUE	Master Protein	A0A5G2QE26	Methyl-CpG-binding protein 2 OS=Sus scrofa OX=9823 GN=MECP2 PE=1 SV=1	15	6	12	6	489	52.4	9.88	37.93	6	1
TRUE	Master Protein	A0A4X1V5U6	Calcium/calmodulin-dependent protein kinase OS=Sus scrofa OX=9823 PE=3 SV=1	21	6	9	4	498	56.3	7.25	36.32	6	1
TRUE	Master Protein	A0A5S6GJG2	Serine/arginine-rich splicing factor 1 OS=Sus scrofa OX=9823 GN=SRSF1 PE=1 SV=1	42	9	13	8	236	26.9	10.29	35.11	9	1
TRUE	Master Protein	A0A287BE52	PWWP domain-containing protein OS=Sus scrofa OX=9823 GN=PSIP1 PE=1 SV=2	17	6	10	6	530	60.1	9.13	34.97	6	1
TRUE	Master Protein	A0A4X1TV43	Creatine kinase OS=Sus scrofa OX=9823 GN=CKB PE=3 SV=1	21	6	10	6	403	44.8	6	33.77	6	1
TRUE	Master Protein	Q6QAQ4	Myosin regulatory light chain (Fragment) OS=Sus scrofa OX=9823 PE=2 SV=1	46	5	11	4	132	15	4.5	33.09	5	1
TRUE	Master Protein	B0LXF7	Aprotinin (Fragment) OS=Sus scrofa OX=9823 PE=4 SV=1	26	1	7	1	58	6.7	7.61	32.41	1	1
TRUE	Master Protein	A0A5K1U8P1	Clathrin heavy chain OS=Sus scrofa OX=9823 GN=CLTC PE=1 SV=1	6	8	10	8	1681	192.6	5.77	32.36	8	1
TRUE	Master Protein	A0A4X1VS48	RNA helicase OS=Sus scrofa OX=9823 GN=DDX17 PE=3 SV=1	19	11	13	6	672	75	8.78	32.33	11	1
TRUE	Master Protein	F1RY92	RRM domain-containing protein OS=Sus scrofa OX=9823 GN=SRSF3 PE=1 SV=2	34	5	10	4	164	19.3	11.65	30.43	5	1
TRUE	Master Protein	A0A480J9N1	Myosin-11 OS=Sus scrofa OX=9823 PE=3 SV=1	7	9	9	6	1931	223.2	5.58	29.09	9	1
TRUE	Master Protein	A0A4X1SG58	Calmodulin OS=Sus scrofa OX=9823 GN=CALM2 PE=3 SV=1	64	4	7	2	83	9.3	4.03	28.64	4	1

TRUE	Master Protein	A0A287AHS0	Calmodulin OS=Sus scrofa OX=9823 GN=CALM3 PE=1 SV=1	35	4	7	2	148	16.7	4.22	28.64	4	1
TRUE	Master Protein	A0A480PNA9	ADP/ATP translocase 2 OS=Sus scrofa OX=9823 PE=3 SV=1	33	8	11	2	298	32.9	9.77	28.08	8	1
TRUE	Master Protein	A0A480TAH1	Myosin-14 isoform 2 OS=Sus scrofa OX=9823 PE=3 SV=1	7	11	11	7	1974	226.5	5.54	27.96	11	1
TRUE	Master Protein	A0A4X1TBF9	ADP/ATP translocase 3 OS=Sus scrofa OX=9823 GN=SLC25A6 PE=3 SV=1	29	7	11	1	297	32.8	9.92	27.4	7	1
TRUE	Master Protein	A0A287B401	RNA-binding protein 14 isoform 1 OS=Sus scrofa OX=9823 GN=RBM14 PE=1 SV=1	14	5	8	5	669	69.5	9.67	27.05	5	1
TRUE	Master Protein	F2Z581	Histone domain-containing protein OS=Sus scrofa OX=9823 PE=1 SV=3	33	5	10	1	85	9.4	10.73	26.85	5	1
TRUE	Master Protein	A0A287ANZ8	Thy-1 antigen OS=Sus scrofa OX=9823 GN=THY1 PE=1 SV=1	29	4	8	4	154	17.2	8.94	26.51	4	1
TRUE	Master Protein	A0A287BSR0	Desmin OS=Sus scrofa OX=9823 GN=DES PE=1 SV=1	7	4	9	1	471	54.1	5.21	25.5	4	1
TRUE	Master Protein	A0A287AN50	Agrin OS=Sus scrofa OX=9823 GN=AGRN PE=1 SV=2	5	8	10	8	2069	218.7	6.27	25.25	8	1
TRUE	Master Protein	A0A480IX08	V-type proton ATPase subunit a OS=Sus scrofa OX=9823 PE=3 SV=1	13	9	9	9	839	96.4	6.57	25.09	9	1
TRUE	Master Protein	A0A287BRP2	Guanine nucleotide-binding protein G(T) subunit alpha-1 isoform X2 OS=Sus scrofa OX=9823 GN=GNAT1 PE=1 SV=1	21	5	8	3	350	40	5.73	24.88	5	1
TRUE	Master Protein	A0A0B8RSH5	Nuclear mitotic apparatus protein 1 OS=Sus scrofa domesticus OX=9825 GN=NUMA1 PE=4 SV=1	6	9	9	9	2088	233.4	5.73	24.2	9	1
TRUE	Master Protein	A0A287BLH5	DEAD box protein 5 OS=Sus scrofa OX=9823 GN=DDX5 PE=3 SV=2	16	8	9	3	617	69.4	9.11	23.03	8	1
TRUE	Master Protein	A0A5G2QCQ9	Calcium/calmodulin-dependent protein kinase OS=Sus scrofa OX=9823 GN=CAMK2B PE=1 SV=1	11	5	7	2	670	72.7	8.12	22.76	5	1
TRUE	Master Protein	A0A4X1TYS6	Elongation factor 1-alpha OS=Sus scrofa OX=9823 GN=EEF1A1 PE=3 SV=1	18	7	7	7	450	49	8.87	21.5	7	1
TRUE	Master Protein	F1SDE3	Centromere protein V OS=Sus scrofa OX=9823 GN=CENPV PE=1 SV=2	29	4	7	4	259	28.1	9.89	21.48	4	1
TRUE	Master Protein	A0A4X1W8E1	WD_REPEATS_REGION domain-containing protein OS=Sus scrofa OX=9823 GN=GNB1 PE=3 SV=1	17	4	6	1	340	37.4	6	21.43	4	1
TRUE	Master Protein	A0A4X1VRD7	FERM domain-containing protein OS=Sus scrofa OX=9823 PE=4 SV=1	7	5	6	5	1196	132.2	5.2	20.8	5	1
TRUE	Master Protein	A0A4X1TYS5	Dynamin GTPase OS=Sus scrofa OX=9823 PE=3 SV=1	12	7	8	7	863	97.1	7.74	20.78	7	1
TRUE	Master Protein	A0A4X1TS37	Heat shock protein HSP 90-alpha OS=Sus scrofa OX=9823 GN=HSP90AA1 PE=3 SV=1	11	7	8	7	789	90.6	5.02	20.47	7	1
TRUE	Master Protein	A0A287A6P1	Actin-depolymerizing factor OS=Sus scrofa OX=9823 GN=GSN PE=3 SV=2	11	5	6	1	780	85.6	6.47	20.15	5	1
TRUE	Master Protein	A0A287AMM6	MICOS complex subunit MIC60 OS=Sus scrofa OX=9823 GN=IMMT PE=1 SV=1	10	5	6	5	746	82.6	6.61	19.68	5	1

TRUE	Master Protein	A0A481B449	Actin-depolymerizing factor OS=Sus scrofa OX=9823 PE=3 SV=1	11	5	6	1	660	71.9	6.73	18.77	5	1
TRUE	Master Protein	A0A4X1UES8	WD_REPEATS_REGION domain-containing protein OS=Sus scrofa OX=9823 GN=GNB2 PE=3 SV=1	17	4	5	1	340	37.3	6	18.7	4	1
TRUE	Master Protein	I3LGB1	Aryl-hydrocarbon-interacting protein-like 1 OS=Sus scrofa OX=9823 GN=AIPL1 PE=1 SV=2	19	5	6	5	328	38.3	6.14	18.67	5	1
TRUE	Master Protein	A0A480YZR2	Cytoskeleton-associated protein 4 OS=Sus scrofa OX=9823 PE=4 SV=1	15	6	6	6	592	64.7	5.73	18.53	6	1
TRUE	Master Protein	A0A480UKG6	Vacuolar proton pump subunit B OS=Sus scrofa OX=9823 PE=3 SV=1	17	6	6	6	511	56.6	5.81	18.16	6	1
TRUE	Master Protein	A0A480PZC9	Calcium/calmodulin-dependent protein kinase OS=Sus scrofa OX=9823 PE=3 SV=1	14	5	6	2	542	60.3	7.17	17.93	5	1
TRUE	Master Protein	K7GSR6	Ecto-5'-nucleotidase OS=Sus scrofa OX=9823 GN=NT5E PE=1 SV=1	12	5	6	5	574	62.8	6.68	17.7	5	1
TRUE	Master Protein	A0A4X1U5K3	S-arrestin OS=Sus scrofa OX=9823 GN=SAG PE=3 SV=1	15	4	6	4	394	43.9	7.11	17.52	4	1
TRUE	Master Protein	A0A287B770	RRM domain-containing protein OS=Sus scrofa OX=9823 GN=SRSF10 PE=1 SV=2	18	5	6	5	261	31.2	11.27	17.16	5	1
TRUE	Master Protein	A0A4X1U9Z4	Nucleoplasmin domain-containing protein OS=Sus scrofa OX=9823 GN=NPM1 PE=3 SV=1	17	3	5	3	259	28.4	4.69	16.98	3	1
TRUE	Master Protein	K7GT43	Structural maintenance of chromosomes protein OS=Sus scrofa OX=9823 GN=SMC1A PE=1 SV=2	6	6	6	6	1187	138	8.72	16.77	6	1
TRUE	Master Protein	A0A4X1ST13	Protein kinase C OS=Sus scrofa OX=9823 GN=PRKCA PE=3 SV=1	12	6	6	2	688	78.7	7.36	15.43	6	1
TRUE	Master Protein	I3LSU1	Non-POU domain containing octamer binding OS=Sus scrofa OX=9823 GN=NONO PE=1 SV=2	14	5	5	5	543	62.4	9.35	15.34	5	1
TRUE	Master Protein	P31006	Guanylate kinase OS=Sus scrofa OX=9823 GN=GUK1 PE=1 SV=2	20	3	5	3	198	21.9	6.95	15.11	3	1
TRUE	Master Protein	A0A287BSC3	Alkaline phosphatase OS=Sus scrofa OX=9823 GN=ALPL PE=1 SV=2	8	5	6	5	834	91.2	8.7	15.06	5	1
TRUE	Master Protein	A0A5S6G3Y8	Heat shock 27 kDa protein OS=Sus scrofa OX=9823 GN=HSPB1 PE=3 SV=1	11	3	5	3	365	40.3	10.55	14.94	3	1
TRUE	Master Protein	A0A286ZPE3	Helix-destabilizing protein OS=Sus scrofa OX=9823 GN=HNRNPA1 PE=1 SV=1	10	3	5	3	373	38.8	9.13	14.89	3	1
TRUE	Master Protein	A0A286ZNE8	VWFA domain-containing protein OS=Sus scrofa OX=9823 GN=CACNA2D2 PE=1 SV=2	4	4	5	4	1068	121.1	5.41	14.25	4	1
TRUE	Master Protein	A0A480W6B7	Tyrosine-protein kinase BAZ1B isoform X1 OS=Sus scrofa OX=9823 PE=4 SV=1	4	4	5	4	1418	162	8.68	14.2	4	1
TRUE	Master Protein	A0A287AGF9	Nop domain-containing protein OS=Sus scrofa OX=9823 GN=NOP58 PE=1 SV=2	8	3	4	3	537	60.4	8.56	13.99	3	1
TRUE	Master Protein	A0A480W0D4	Heterogeneous nuclear ribonucleoprotein A3 isoform a OS=Sus scrofa OX=9823 PE=4 SV=1	20	5	5	5	378	39.6	9.01	13.62	5	1
TRUE	Master Protein	P62272	40S ribosomal protein S18 OS=Sus scrofa OX=9823 GN=RPS18 PE=1 SV=3	30	5	6	5	152	17.7	10.99	13.53	5	1

TRUE	Master Protein	A0A4X1V3C3	Small nuclear ribonucleoprotein Sm D3 OS=Sus scrofa OX=9823 GN=SNRPD3 PE=3 SV=1	31	3	4	3	126	13.9	10.32	13.26	3	1
TRUE	Master Protein	A0A0B8RSA0	Solute carrier family 25 (Mitochondrial carrier: glutamate), member 22 OS=Sus scrofa domesticus OX=9825 GN=SLC25A22 PE=3 SV=1	16	5	5	5	322	34.4	9.54	13.03	5	1
TRUE	Master Protein	A0A4X1U434	Peptidylprolyl isomerase OS=Sus scrofa OX=9823 PE=4 SV=1	7	3	4	3	604	63.7	7.05	12.99	3	1
TRUE	Master Protein	A0A4X1TWR8	H15 domain-containing protein OS=Sus scrofa OX=9823 GN=LOC100516295 PE=3 SV=1	18	4	5	4	214	22.5	10.71	12.92	4	1
TRUE	Master Protein	A0A481A9M2	IgLON family member 5 OS=Sus scrofa OX=9823 GN=LOC110261043 PE=4 SV=1	19	5	5	5	348	38.2	6.76	12.68	5	1
TRUE	Master Protein	A0A5G2QFH4	RRM domain-containing protein OS=Sus scrofa OX=9823 GN=TRA2B PE=1 SV=1	16	2	4	2	188	21.9	10.15	12.63	2	1
TRUE	Master Protein	A0A480W7D9	Telomeric repeat-binding factor 2 isoform X2 OS=Sus scrofa OX=9823 PE=4 SV=1	11	3	4	3	288	31.7	9.54	12.51	3	1
TRUE	Master Protein	Q9MZ15	Voltage-dependent anion-selective channel protein 2 OS=Sus scrofa OX=9823 GN=VDAC2 PE=2 SV=1	20	4	4	4	294	31.6	7.56	12.41	4	1
TRUE	Master Protein	A0A480JVL5	RNA-binding protein with serine-rich domain 1 OS=Sus scrofa OX=9823 GN=RNPS1 PE=1 SV=1	8	2	4	2	305	34.2	11.84	12.39	2	1
TRUE	Master Protein	A0A480Y9K7	Opioid-binding protein/cell adhesion molecule isoform b OS=Sus scrofa OX=9823 PE=4 SV=1	22	4	4	3	345	38	6.77	12.31	4	1
TRUE	Master Protein	K9J4L8	Protein kinase C OS=Sus scrofa OX=9823 GN=PRKCB_tv2 PE=1 SV=1	10	5	5	1	673	76.9	7.02	12.28	5	1
TRUE	Master Protein	A0A5G2QFK1	RNA helicase OS=Sus scrofa OX=9823 GN=EIF4A3 PE=3 SV=1	11	3	4	3	390	44.5	6.47	12.18	3	1
TRUE	Master Protein	A0A0B8RTL5	Heterogeneous nuclear ribonucleoprotein R OS=Sus scrofa domesticus OX=9825 GN=HNRNPR PE=4 SV=1	5	2	4	2	532	59.6	9.16	12.17	2	1
TRUE	Master Protein	A0A4X1SKZ9	VWFA domain-containing protein OS=Sus scrofa OX=9823 GN=CACNA2D1 PE=3 SV=1	5	4	5	4	1084	122.3	5.2	12.04	4	1
TRUE	Master Protein	A0A4X1TSA4	Protein unc-119 homolog A OS=Sus scrofa OX=9823 GN=UNC119 PE=3 SV=1	17	3	4	3	240	26.9	6.15	11.93	3	1
TRUE	Master Protein	A0A480W9G4	40S ribosomal protein S13 OS=Sus scrofa OX=9823 PE=3 SV=1	20	3	4	3	148	16.9	10.45	11.86	3	1
TRUE	Master Protein	A0A287BRY0	Tropomyosin alpha-1 chain OS=Sus scrofa OX=9823 GN=TPM1 PE=1 SV=2	19	4	5	2	248	28.7	4.77	11.68	4	1
TRUE	Master Protein	A0A4X1TGS8	Myosin regulatory light polypeptide 9 OS=Sus scrofa OX=9823 GN=MYL9 PE=4 SV=1	12	2	5	1	172	19.8	4.82	11.65	2	1
TRUE	Master Protein	A0A5G2RGK0	Purine rich element binding protein A OS=Sus scrofa OX=9823 GN=PURA PE=1 SV=1	12	3	3	3	482	50.5	8.43	11.62	3	1
TRUE	Master Protein	A0A4X1V3Y1	SAP domain-containing protein OS=Sus scrofa OX=9823 GN=ACIN1 PE=4 SV=1	4	4	5	4	1252	141.4	6.14	11.4	4	1
TRUE	Master Protein	A0A4X1UWL5	Cellular retinoic acid-binding protein 1 OS=Sus scrofa OX=9823 GN=CRABP1 PE=3 SV=1	53	4	4	4	110	12.5	5.3	11.32	4	1
TRUE	Master Protein	A0A4X1UGM9	SEC7 domain-containing protein OS=Sus scrofa OX=9823 PE=3 SV=1	4	4	5	4	1203	129	6.55	11.17	4	1

TRUE	Master Protein	F1RN35	G protein-coupled receptor kinase OS=Sus scrofa OX=9823 GN=GRK1 PE=1 SV=1	5	2	4	2	564	63.3	5.78	11.17	2	1
TRUE	Master Protein	P46410	Glutamine synthetase OS=Sus scrofa OX=9823 GN=GLUL PE=2 SV=3	13	5	5	5	373	42	6.74	11.07	5	1
TRUE	Master Protein	O62680	CD59 glycoprotein OS=Sus scrofa OX=9823 GN=CD59 PE=1 SV=1	9	1	3	1	123	13.8	7.94	10.94	1	1
TRUE	Master Protein	A0A4X1T5Y2	High mobility group AT-hook protein 1 OS=Sus scrofa OX=9823 GN=HMGA1 PE=3 SV=1	15	1	3	1	107	11.6	10.32	10.91	1	1
TRUE	Master Protein	A0A287B5N7	Tropomyosin alpha-1 chain OS=Sus scrofa OX=9823 GN=TPM1 PE=1 SV=2	11	3	4	1	351	40	4.75	10.85	3	1
TRUE	Master Protein	A0A5G2QMG4	Unconventional myosin-6 OS=Sus scrofa OX=9823 GN=MYO6 PE=1 SV=1	4	4	4	4	1226	141.6	8.44	10.83	4	1
TRUE	Master Protein	A0A480SLY1	Tropomyosin alpha-3 chain isoform Tpm3.3cy OS=Sus scrofa OX=9823 PE=3 SV=1	16	3	4	1	248	28.7	4.72	10.79	3	1
TRUE	Master Protein	A1XQS3	Mitochondrial NDUFA4 OS=Sus scrofa OX=9823 GN=NDUFA4 PE=2 SV=1	28	2	4	2	82	9.3	9.38	10.51	2	1
TRUE	Master Protein	A0A4X1U2F8	Histone deacetylase complex subunit SAP18 OS=Sus scrofa OX=9823 GN=SAP18 PE=3 SV=1	23	2	3	2	153	17.6	9.35	10.4	2	1
TRUE	Master Protein	A0A4X1TV68	Signal recognition particle receptor subunit beta OS=Sus scrofa OX=9823 PE=3 SV=1	17	3	4	3	271	29.9	9.33	10.25	3	1
TRUE	Master Protein	Q0Z8U2	40S ribosomal protein S3 OS=Sus scrofa OX=9823 GN=RPS3 PE=1 SV=1	16	3	4	3	243	26.7	9.66	10.23	3	1
TRUE	Master Protein	A0A287BBY5	RNA helicase OS=Sus scrofa OX=9823 GN=DHX15 PE=1 SV=1	5	4	4	4	766	87.8	7.24	9.63	4	1
TRUE	Master Protein	A1XQU3	60S ribosomal protein L14 OS=Sus scrofa OX=9823 GN=RPL14 PE=1 SV=1	6	1	3	1	213	23.3	10.77	9.4	1	1
TRUE	Master Protein	A0A287AV47	PWI domain-containing protein OS=Sus scrofa OX=9823 GN=SRRM1 PE=1 SV=1	4	1	2	1	968	109.3	11.71	9.31	1	1
TRUE	Master Protein	I3L8C5	Heat shock 70 kDa protein 12A isoform 2 OS=Sus scrofa OX=9823 GN=HSPA12A PE=1 SV=1	7	4	4	4	675	74.8	6.61	9.27	4	1
TRUE	Master Protein	Q0QEV2	Ribosomal protein L18 (Fragment) OS=Sus scrofa OX=9823 GN=RPL18 PE=2 SV=1	15	2	3	2	166	18.9	11.78	9.2	2	1
TRUE	Master Protein	A0A4X1W8J3	TAR DNA-binding protein 43 OS=Sus scrofa OX=9823 PE=4 SV=1	10	3	3	3	435	47.2	7.31	9.17	3	1
TRUE	Master Protein	A0A4X1SVK7	Repressor/activator protein 1 homolog OS=Sus scrofa OX=9823 PE=3 SV=1	10	3	3	3	401	44.3	4.77	9.16	3	1
TRUE	Master Protein	A0A5G2R0B1	Hexokinase OS=Sus scrofa OX=9823 GN=HK1 PE=1 SV=1	5	4	4	4	779	86.7	7.28	9.15	4	1
TRUE	Master Protein	A0A4X1UX38	Voltage-dependent anion-selective channel protein 1 OS=Sus scrofa OX=9823 GN=VDAC1 PE=3 SV=1	14	4	4	3	302	33	8.69	8.99	4	1
TRUE	Master Protein	A0A287BFM2	SAM domain-containing protein OS=Sus scrofa OX=9823 GN=SAMD11 PE=1 SV=2	6	3	3	3	683	72.9	7.33	8.98	3	1
TRUE	Master Protein	A0A4X1TI48	Nucleolin OS=Sus scrofa OX=9823 GN=NCL PE=4 SV=1	3	2	3	2	699	75.2	4.63	8.92	2	1

TRUE	Master Protein	A0A4X1VYE4	60S ribosomal protein L13a OS=Sus scrofa OX=9823 GN=RPL13A PE=3 SV=1	12	2	3	2	204	23.7	11.09	8.85	2	1
TRUE	Master Protein	I3LDH3	Poly [ADP-ribose] polymerase OS=Sus scrofa OX=9823 GN=PARP1 PE=1 SV=3	5	4	4	4	1017	113.1	8.87	8.82	4	1
TRUE	Master Protein	A0A4X1W191	DZF domain-containing protein OS=Sus scrofa OX=9823 GN=ILF2 PE=4 SV=1	12	3	3	3	390	43	5.26	8.77	3	1
TRUE	Master Protein	P00355	Glyceraldehyde-3-phosphate dehydrogenase OS=Sus scrofa OX=9823 GN=GAPDH PE=1 SV=4	15	3	3	3	333	35.8	8.35	8.72	3	1
TRUE	Master Protein	Q06AS7	GBI1 OS=Sus scrofa OX=9823 PE=2 SV=1	8	2	3	1	355	40.5	5.38	8.5	2	1
TRUE	Master Protein	A0A480RU92	Aspartate--tRNA ligase, cytoplasmic OS=Sus scrofa OX=9823 PE=3 SV=1	8	3	3	3	482	55	5.87	8.46	3	1
TRUE	Master Protein	A0A287A4F2	Splicing factor, proline-and glutamine-rich isoform X1 OS=Sus scrofa OX=9823 GN=SFPQ PE=4 SV=1	5	3	3	3	702	75.5	9.44	8.33	3	1
TRUE	Master Protein	A0A287AJQ2	Phosphoglycerate mutase OS=Sus scrofa OX=9823 GN=PGAM1 PE=1 SV=1	16	3	3	3	254	28.8	7.18	8.14	3	1
TRUE	Master Protein	A0A5S6HMS9	RNA-binding protein 4B OS=Sus scrofa OX=9823 GN=RBM4B PE=1 SV=1	11	3	3	1	369	41.1	6.8	8.02	3	1
TRUE	Master Protein	A0A5G2QD67	Galectin OS=Sus scrofa OX=9823 GN=LGALS1 PE=1 SV=1	15	2	3	2	164	17.9	5.05	8.01	2	1
TRUE	Master Protein	A0A287AY86	Microtubule-associated protein OS=Sus scrofa OX=9823 GN=MAP4 PE=1 SV=1	2	3	3	3	2220	235.7	6.02	7.99	3	1
TRUE	Master Protein	A0A4X1W115	Small nuclear ribonucleoprotein Sm D2 OS=Sus scrofa OX=9823 GN=SNRPD2 PE=3 SV=1	16	2	3	2	118	13.5	9.91	7.89	2	1
TRUE	Master Protein	A0A480LV60	E3 SUMO-protein ligase RanBP2 isoform X6 OS=Sus scrofa OX=9823 PE=4 SV=1	1	3	3	3	3132	346.4	6.23	7.7	3	1
TRUE	Master Protein	Q197W4	Cyclin-dependent kinase 5 OS=Sus scrofa OX=9823 GN=CDK5 PE=1 SV=2	8	2	3	2	292	33.3	7.66	7.38	2	1
TRUE	Master Protein	A0A287AFC3	ELAV-like protein OS=Sus scrofa OX=9823 GN=ELAVL1 PE=1 SV=1	11	3	3	3	303	33.5	8.24	7.32	3	1
TRUE	Master Protein	A0A480VB01	U2 snRNP-associated SURP motif-containing protein isoform 2 OS=Sus scrofa OX=9823 PE=4 SV=1	4	3	3	3	1028	118.1	8.47	7.31	3	1
TRUE	Master Protein	F1RUT1	RNA-binding protein 4 isoform 1 OS=Sus scrofa OX=9823 GN=RBM14 PE=1 SV=3	11	3	3	1	365	40.4	7.08	7.23	3	1
TRUE	Master Protein	F2Z5F5	40S ribosomal protein S8 OS=Sus scrofa OX=9823 GN=RPS8 PE=1 SV=3	11	2	3	2	208	24.2	10.32	7.18	2	1
TRUE	Master Protein	A0A4X1UJS8	V-type proton ATPase subunit OS=Sus scrofa OX=9823 GN=ATP6V0D1 PE=3 SV=1	11	3	3	3	351	40.3	5	7.11	3	1
TRUE	Master Protein	A0A0B8RZ10	Ubiquitin-activating enzyme E1 OS=Sus scrofa domesticus OX=9825 GN=UBA1 PE=3 SV=1	3	2	2	2	1058	117.7	5.85	7.1	2	1
TRUE	Master Protein	A0A5G2R7S1	LEM domain-containing protein OS=Sus scrofa OX=9823 GN=EMD PE=4 SV=1	7	1	2	1	224	24.9	4.87	7.05	1	1
TRUE	Master Protein	A0A481C3R6	Pre-mRNA-processing-splicing factor 8 isoform X1 OS=Sus scrofa OX=9823 PE=4 SV=1	2	2	2	2	1649	194.8	9.14	6.97	2	1

TRUE	Master Protein	A0A4X1VFI7	ADP-ribosyl cyclase/cyclic ADP-ribose hydrolase OS=Sus scrofa OX=9823 PE=3 SV=1	11	2	2	2	289	32.1	6.3	6.95	2	1
TRUE	Master Protein	A0A4X1VQM3	Clathrin light chain OS=Sus scrofa OX=9823 PE=3 SV=1	14	2	2	2	211	23.1	4.53	6.91	2	1
TRUE	Master Protein	A0A4X1SJ12	Nop domain-containing protein OS=Sus scrofa OX=9823 GN=NOP56 PE=3 SV=1	6	3	3	3	596	66.1	9.36	6.85	3	1
TRUE	Master Protein	A0A480V6N0	Double-stranded RNA-specific adenosine deaminase isoform X5 OS=Sus scrofa OX=9823 PE=4 SV=1	4	2	2	2	617	68.9	9.38	6.76	2	1
TRUE	Master Protein	Q29380	Voltage-dependent anion-selective channel protein 3 OS=Sus scrofa OX=9823 GN=VDAC3 PE=2 SV=2	10	2	2	1	283	30.6	8.66	6.71	2	1
TRUE	Master Protein	F1SIT7	60S acidic ribosomal protein P1 OS=Sus scrofa OX=9823 GN=RPLP1 PE=1 SV=1	29	2	2	2	114	11.5	4.32	6.57	2	1
TRUE	Master Protein	A0A5G2R2A7	60S ribosomal protein L4 OS=Sus scrofa OX=9823 GN=RPL4 PE=1 SV=1	7	2	2	2	417	47.2	10.8	6.49	2	1
TRUE	Master Protein	F1SA23	Retinol dehydrogenase 12 OS=Sus scrofa OX=9823 GN=RDH12 PE=1 SV=2	11	3	3	3	316	35.1	9.63	6.48	3	1
TRUE	Master Protein	A0A287A014	40S ribosomal protein S15a OS=Sus scrofa OX=9823 GN=RPS15A PE=1 SV=1	26	3	3	3	100	11.5	10.15	6.48	3	1
TRUE	Master Protein	A0A480YVB6	RNA helicase OS=Sus scrofa OX=9823 PE=4 SV=1	5	2	2	2	508	57.6	5.99	5.95	2	1
TRUE	Master Protein	A0A287AUY5	PWWP domain-containing protein OS=Sus scrofa OX=9823 GN=HDGFL3 PE=1 SV=1	8	1	2	1	203	22.6	7.99	5.85	1	1
TRUE	Master Protein	A0A4X1VUP5	PHB domain-containing protein OS=Sus scrofa OX=9823 GN=ERLIN2 PE=3 SV=1	7	2	2	2	363	40.2	6.2	5.84	2	1
TRUE	Master Protein	A0A480WC71	Band 4.1-like protein 2 isoform X7 OS=Sus scrofa OX=9823 PE=4 SV=1	4	2	2	2	865	96.4	5.58	5.83	2	1
TRUE	Master Protein	A0A4X1UZN2	V-type proton ATPase subunit G OS=Sus scrofa OX=9823 GN=ATP6V1G1 PE=3 SV=1	14	1	2	1	118	13.7	8.43	5.72	1	1
TRUE	Master Protein	A0A480HRR5	Elongation factor Tu OS=Sus scrofa OX=9823 PE=3 SV=1	5	2	2	2	566	61.3	8.32	5.71	2	1
TRUE	Master Protein	A0A480EKD7	Kin of IRRE-like protein 1 isoform X1 OS=Sus scrofa OX=9823 GN=KIRREL1 PE=4 SV=1	5	1	1	1	773	84.9	5.67	5.63	1	1
TRUE	Master Protein	A0A4X1UE92	Spectrin beta chain OS=Sus scrofa OX=9823 GN=SPTB PE=3 SV=1	1	2	2	1	2340	269.7	5.39	5.53	2	1
TRUE	Master Protein	A0A4X1W661	CSD_1 domain-containing protein OS=Sus scrofa OX=9823 GN=YBX1 PE=4 SV=1	19	2	2	2	324	35.9	9.88	5.52	2	1
TRUE	Master Protein	F1S564	RNA 3'-terminal phosphate cyclase OS=Sus scrofa OX=9823 GN=RTCA PE=1 SV=3	5	1	1	1	366	39.2	8.09	5.51	1	1
TRUE	Master Protein	A0A4X1VUZ7	Glutathione peroxidase OS=Sus scrofa OX=9823 GN=GPX4 PE=3 SV=1	15	2	2	2	197	22.2	8.31	5.49	2	1
TRUE	Master Protein	F1RQ90	RNA-binding motif protein, X chromosome isoform 1 OS=Sus scrofa OX=9823 GN=RBMX PE=1 SV=1	6	2	2	2	390	42.2	10.05	5.48	2	1
TRUE	Master Protein	Q9GL64	Transcription factor A (Fragment) OS=Sus scrofa OX=9823 GN=Tfam PE=2 SV=1	7	1	2	1	149	17.9	9.32	5.48	1	1

TRUE	Master Protein	A1XQU9	40S ribosomal protein S20 OS=Sus scrofa OX=9823 GN=RPS20 PE=1 SV=1	15	2	2	2	119	13.3	9.94	5.42	2	1
TRUE	Master Protein	A0A287AV76	BAG domain-containing protein OS=Sus scrofa OX=9823 GN=BAG2 PE=1 SV=1	13	2	2	2	210	23.6	6.38	5.34	2	1
TRUE	Master Protein	A0A4X1TDZ5	Alpha(B)-crystallin OS=Sus scrofa OX=9823 GN=CRYAB PE=3 SV=1	9	2	2	2	237	27.6	9.73	5.28	2	1
TRUE	Master Protein	A0A5G2QDJ2	Polyubiquitin-C OS=Sus scrofa OX=9823 GN=UBC PE=4 SV=1	37	2	2	2	837	94.1	7.68	5.23	2	1
TRUE	Master Protein	A0A480PH82	HIV Tat-specific factor 1 OS=Sus scrofa OX=9823 PE=3 SV=1	4	1	1	1	788	87.2	4.45	5.19	1	1
TRUE	Master Protein	A0A480KS67	FACT complex subunit SSRP1 OS=Sus scrofa OX=9823 PE=3 SV=1	5	2	2	2	655	75.2	7.53	5.17	2	1
TRUE	Master Protein	Q2YGT9	60S ribosomal protein L6 OS=Sus scrofa OX=9823 GN=RPL6 PE=1 SV=3	9	2	2	2	284	32.2	10.73	5.14	2	1
TRUE	Master Protein	A0A480IZZ5	Transcription activator BRG1 isoform X6 OS=Sus scrofa OX=9823 PE=3 SV=1	2	2	2	2	1617	181.6	8.07	5.14	2	1
TRUE	Master Protein	A0A287BKP9	von Willebrand factor A domain-containing protein 1 isoform 1 OS=Sus scrofa OX=9823 GN=VWA1 PE=1 SV=1	6	2	2	2	452	47.7	10.07	5.11	2	1
TRUE	Master Protein	A0A2C9F345	U1 small nuclear ribonucleoprotein A OS=Sus scrofa OX=9823 GN=SNRPA PE=3 SV=1	10	2	2	2	281	31.2	9.83	5	2	1
TRUE	Master Protein	F1S3F6	Ceh-10 homeodomain-containing homolog OS=Sus scrofa OX=9823 GN=VSX2 PE=1 SV=3	4	1	2	1	361	39.3	6.96	4.93	1	1
TRUE	Master Protein	A0A287BP50	PITH domain-containing protein OS=Sus scrofa OX=9823 GN=TXNL1 PE=1 SV=1	8	1	1	1	285	31.9	4.89	4.91	1	1
TRUE	Master Protein	I3LH70	LAM_G_DOMAIN domain-containing protein OS=Sus scrofa OX=9823 GN=COL18A1 PE=1 SV=3	1	2	2	2	1613	162.8	8.28	4.9	2	1
TRUE	Master Protein	A0A480VKU0	Laminin subunit alpha-5 isoform X1 OS=Sus scrofa OX=9823 PE=4 SV=1	1	2	2	2	3484	374.4	6.81	4.89	2	1
TRUE	Master Protein	F1DFL7	Aminopeptidase OS=Sus scrofa OX=9823 PE=2 SV=1	3	2	2	2	963	108.8	5.25	4.78	2	1
TRUE	Master Protein	A0A287AKP9	RRM domain-containing protein OS=Sus scrofa OX=9823 GN=RALY PE=1 SV=1	5	2	2	2	365	37.9	9.28	4.78	2	1
TRUE	Master Protein	A0A481DHP5	40S ribosomal protein S25 (Fragment) OS=Sus scrofa OX=9823 PE=3 SV=1	14	2	2	2	138	14.9	10.11	4.75	2	1
TRUE	Master Protein	F1SFF4	RNA transcription, translation and transport factor protein OS=Sus scrofa OX=9823 GN=RTRAF PE=1 SV=1	7	1	1	1	244	28	6.65	4.75	1	1
TRUE	Master Protein	A0A480J4Z3	Ubiquitin carboxyl-terminal hydrolase OS=Sus scrofa OX=9823 PE=3 SV=1	3	2	2	2	835	93.4	5.1	4.73	2	1
TRUE	Master Protein	A0A4X1T8H6	Phosphodiesterase OS=Sus scrofa OX=9823 PE=3 SV=1	2	2	2	2	862	99.6	5.77	4.67	2	1
TRUE	Master Protein	A0A4X1UVB3	Protein lin-7 homolog OS=Sus scrofa OX=9823 GN=LIN7A PE=3 SV=1	8	2	2	2	233	26	8.72	4.66	2	1
TRUE	Master Protein	A0A4X1VR00	Dynamin-1-like protein OS=Sus scrofa OX=9823 GN=DNM1L PE=3 SV=1	4	2	2	2	725	80.5	6.71	4.63	2	1

TRUE	Master Protein	A0A480VGI2	DNA helicase OS=Sus scrofa OX=9823 PE=3 SV=1	2	2	2	2	1220	138.9	7.06	4.6	2	1
TRUE	Master Protein	I3LSE4	Protein kinase domain-containing protein OS=Sus scrofa OX=9823 GN=CAMKV PE=1 SV=3	5	2	2	2	559	60.5	6.49	4.56	2	1
TRUE	Master Protein	A0A287AVQ1	RNA helicase OS=Sus scrofa OX=9823 GN=DDX3X PE=1 SV=1	3	2	2	1	659	72.9	7.59	4.56	2	1
TRUE	Master Protein	A0A480TW29	Serine/arginine-rich splicing factor 2 (Fragment) OS=Sus scrofa OX=9823 PE=3 SV=1	8	2	2	2	211	24.4	11.97	4.5	2	1
TRUE	Master Protein	I3LQX0	SUN domain-containing protein OS=Sus scrofa OX=9823 GN=SUN1 PE=1 SV=3	3	2	2	2	717	77.9	7.05	4.47	2	1
TRUE	Master Protein	A0A5G2RAE1	RNA helicase OS=Sus scrofa OX=9823 GN=DDX46 PE=1 SV=1	2	2	2	2	1032	117.3	9.29	4.46	2	1
TRUE	Master Protein	A0A480TAZ5	SWI/SNF-related matrix-associated actin-dependent regulator of chromatin subfamily A member 5 OS=Sus scrofa OX=9823 PE=3 SV=1	2	2	2	2	1052	121.8	8.31	4.46	2	1
TRUE	Master Protein	A0A287BGK8	Fibroblast growth factor OS=Sus scrofa OX=9823 GN=FGF2 PE=1 SV=1	6	2	2	2	316	33.6	11.4	4.4	2	1
TRUE	Master Protein	A0A480QSC0	Amino acid transporter OS=Sus scrofa OX=9823 PE=3 SV=1	3	1	1	1	447	49.6	9.54	4.38	1	1
TRUE	Master Protein	A0A5G2R4Z1	Sorting nexin-3 OS=Sus scrofa OX=9823 GN=SNX3 PE=1 SV=1	13	2	2	2	162	18.8	8.66	4.27	2	1
TRUE	Master Protein	A0A287AKE5	Protein lin-7 homolog OS=Sus scrofa OX=9823 GN=LIN7C PE=1 SV=2	10	2	2	2	199	22	7.85	4.23	2	1
TRUE	Master Protein	A0A5G2QQC6	Mediator of DNA damage checkpoint protein 1 OS=Sus scrofa OX=9823 GN=MDC1 PE=4 SV=1	1	1	1	1	2047	218.5	5.45	4.23	1	1
TRUE	Master Protein	A0A480TIY8	General transcription factor 3C polypeptide 5 isoform 2 OS=Sus scrofa OX=9823 PE=4 SV=1	4	2	2	2	513	58.7	7.03	4.2	2	1
TRUE	Master Protein	A0A4X1UA50	Nucleoporin_C domain-containing protein OS=Sus scrofa OX=9823 GN=NUP133 PE=3 SV=1	2	1	1	1	1141	127.3	5.21	4.18	1	1
TRUE	Master Protein	A0A287AGK7	DNA helicase OS=Sus scrofa OX=9823 GN=CHD2 PE=4 SV=1	1	2	2	2	1781	205.5	7.53	4.16	2	1
TRUE	Master Protein	A0A4X1UFA1	Ribosomal protein L19 OS=Sus scrofa OX=9823 PE=3 SV=1	14	2	2	2	181	21.4	11.84	4.15	2	1
TRUE	Master Protein	A0A287B0T7	Chromobox protein homolog 3 isoform X1 OS=Sus scrofa OX=9823 GN=CBX3 PE=1 SV=1	14	2	2	2	183	20.8	5.33	4.14	2	1
TRUE	Master Protein	A0A4X1WBN0	IF rod domain-containing protein OS=Sus scrofa OX=9823 PE=3 SV=1	3	2	2	1	565	60.5	8.22	4.11	2	1
TRUE	Master Protein	F1RQ91	40S ribosomal protein S4 OS=Sus scrofa OX=9823 GN=RPS4X PE=1 SV=3	5	1	1	1	242	27.6	10.08	4.06	1	1
TRUE	Master Protein	A0A480N4X7	40S ribosomal protein S26 OS=Sus scrofa OX=9823 PE=3 SV=1	14	1	1	1	107	12.2	10.83	3.95	1	1
TRUE	Master Protein	A0A287A4C0	DUF3504 domain-containing protein OS=Sus scrofa OX=9823 GN=QRICH1 PE=4 SV=1	2	1	1	1	779	86.8	5.95	3.94	1	1
TRUE	Master Protein	A0A4X1TH39	DZF domain-containing protein OS=Sus scrofa OX=9823 GN=ZFR PE=4 SV=1	2	1	1	1	1003	109.4	9.23	3.89	1	1

TRUE	Master Protein	A0A5G2QJ52	14_3_3 domain-containing protein OS=Sus scrofa OX=9823 GN=CRK PE=1 SV=1	5	1	1	1	266	30.5	6.43	3.72	1	1
TRUE	Master Protein	D3K5K3	DNA mismatch repair protein OS=Sus scrofa OX=9823 GN=MSH2 PE=2 SV=1	2	1	1	1	934	104.5	5.64	3.7	1	1
TRUE	Master Protein	A0A287AC97	MICOS complex subunit MIC19 isoform 2 OS=Sus scrofa OX=9823 GN=CHCHD3 PE=1 SV=1	6	1	1	1	235	27.2	8.19	3.7	1	1
TRUE	Master Protein	A0A480KG54	Ciliary neurotrophic factor receptor subunit alpha OS=Sus scrofa OX=9823 PE=3 SV=1	6	1	1	1	372	40.9	6.95	3.68	1	1
TRUE	Master Protein	F1RLT6	Guanine nucleotide-binding protein G(Z) subunit alpha OS=Sus scrofa OX=9823 GN=GNAZ PE=1 SV=1	4	1	1	1	355	40.7	7.61	3.65	1	1
TRUE	Master Protein	Q29375	60S ribosomal protein L7a (Fragment) OS=Sus scrofa OX=9823 GN=RPL7A PE=2 SV=1	11	1	1	1	132	14.6	10.14	3.56	1	1
TRUE	Master Protein	A0A4X1TVR0	DNA-directed RNA polymerase subunit beta OS=Sus scrofa OX=9823 PE=3 SV=1	2	1	1	1	1196	135.9	7.88	3.54	1	1
TRUE	Master Protein	A0A480L1A5	Regulator of microtubule dynamics protein 3 isoform 1 OS=Sus scrofa OX=9823 PE=4 SV=1	3	1	1	1	496	54.5	5.47	3.53	1	1
TRUE	Master Protein	A0A5G2QTJ8	Importin subunit alpha OS=Sus scrofa OX=9823 GN=KPNA1 PE=1 SV=1	3	1	1	1	531	59.4	4.97	3.47	1	1
TRUE	Master Protein	A0A480V6Z4	ATP synthase subunit alpha, mitochondrial isoform a OS=Sus scrofa OX=9823 PE=3 SV=1	5	1	1	1	323	34.6	9.44	3.46	1	1
TRUE	Master Protein	F1SQA9	GATOR complex protein SEC13 OS=Sus scrofa OX=9823 GN=SEC13 PE=1 SV=4	5	1	1	1	333	36.7	5.48	3.44	1	1
TRUE	Master Protein	F1SUT1	Pleckstrin homology domain-containing family B member 1 isoform a OS=Sus scrofa OX=9823 GN=PLEKHB1 PE=1 SV=3	10	1	1	1	224	25.4	8.46	3.38	1	1
TRUE	Master Protein	A0A5G2Q7N6	60S ribosomal protein L11 OS=Sus scrofa OX=9823 GN=RPL11 PE=3 SV=1	7	1	1	1	192	22	9.72	3.24	1	1
TRUE	Master Protein	A0A287AHC5	Guanine nucleotide-binding protein subunit gamma OS=Sus scrofa OX=9823 PE=3 SV=1	21	1	1	1	68	7.5	8.51	3.24	1	1
TRUE	Master Protein	P01965	Hemoglobin subunit alpha OS=Sus scrofa OX=9823 GN=HBA PE=1 SV=1	11	1	1	1	141	15	8.7	3.19	1	1
TRUE	Master Protein	F2YI04	Fas apoptosis inhibitory molecule 1 OS=Sus scrofa OX=9823 GN=FAIM1 PE=2 SV=1	8	1	1	1	200	22.5	5.02	3.18	1	1
TRUE	Master Protein	A0A287B8N1	ATP-dependent 6-phosphofructokinase OS=Sus scrofa OX=9823 GN=PFKM PE=1 SV=1	3	1	1	1	809	88.9	8.34	3.18	1	1
TRUE	Master Protein	A0A4X1W398	AAA domain-containing protein OS=Sus scrofa OX=9823 GN=LOC100525876 PE=3 SV=1	2	1	1	1	493	56.3	9.5	3.15	1	1
TRUE	Master Protein	A0A480V0Y7	Metastasis-associated protein MTA1 isoform MTA1s OS=Sus scrofa OX=9823 PE=4 SV=1	2	1	1	1	429	48.7	5.5	3.14	1	1
TRUE	Master Protein	A0A480TKG0	Nuclear pore complex protein Nup155 isoform 1 OS=Sus scrofa OX=9823 PE=3 SV=1	1	1	1	1	1392	155.2	6.3	3.11	1	1
TRUE	Master Protein	I3LK59	2-phospho-D-glycerate hydro-lyase OS=Sus scrofa OX=9823 GN=ENO1 PE=1 SV=3	4	1	1	1	434	47.1	7.14	3.1	1	1
TRUE	Master Protein	A0A4X1U612	ANK_REP_REGION domain-containing protein OS=Sus scrofa OX=9823 GN=ANKRD10 PE=4 SV=1	14	1	1	1	161	17	6.77	3.07	1	1

TRUE	Master Protein	A0A4X1T7A1	RRM domain-containing protein OS=Sus scrofa OX=9823 GN=MSI1 PE=3 SV=1	5	1	1	1	311	33.5	9.17	3.02	1	1
TRUE	Master Protein	F2Z594	High mobility group protein B1 OS=Sus scrofa OX=9823 GN=HMGB1 PE=1 SV=3	5	1	1	1	220	25.3	9.95	3.01	1	1
TRUE	Master Protein	A0A481B6W1	Peptidyl-prolyl cis-trans isomerase OS=Sus scrofa OX=9823 PE=3 SV=1	3	1	1	1	492	57.2	5.94	3.01	1	1
TRUE	Master Protein	A0A4X1U3M8	Mitochondrial fission factor OS=Sus scrofa OX=9823 GN=MFF PE=3 SV=1	6	1	1	1	237	27	6.62	2.98	1	1
TRUE	Master Protein	A0A480LR40	Tricarboxylate transport protein, mitochondrial isoform a (Fragment) OS=Sus scrofa OX=9823 PE=3 SV=1	3	1	1	1	395	42.8	10.46	2.98	1	1
TRUE	Master Protein	A0A5G2R2K0	RNA helicase aquarius OS=Sus scrofa OX=9823 GN=AQR PE=1 SV=1	1	1	1	1	1485	170.7	6.4	2.95	1	1
TRUE	Master Protein	A0A4X1UDF8	60S ribosomal protein L32 OS=Sus scrofa OX=9823 GN=RPL32 PE=3 SV=1	7	1	1	1	149	17.5	11.05	2.94	1	1
TRUE	Master Protein	A0A480PKL9	Prospero homeobox protein 1 isoform X2 OS=Sus scrofa OX=9823 PE=4 SV=1	3	1	1	1	607	67.6	7.83	2.92	1	1
TRUE	Master Protein	A0A481D0A9	Biglycan OS=Sus scrofa OX=9823 PE=3 SV=1	3	1	1	1	488	53.3	9.06	2.88	1	1
TRUE	Master Protein	A0A4X1VZ98	V-type proton ATPase subunit F OS=Sus scrofa OX=9823 PE=3 SV=1	10	1	1	1	118	13.2	5.3	2.88	1	1
TRUE	Master Protein	Q19PY3	RNA-splicing ligase RtcB homolog OS=Sus scrofa OX=9823 GN=RTCB PE=2 SV=1	2	1	1	1	505	55.2	7.23	2.88	1	1
TRUE	Master Protein	F1RWX5	ATPase WRNIP1 isoform 1 OS=Sus scrofa OX=9823 GN=WRNIP1 PE=1 SV=2	2	1	1	1	638	69	6.34	2.88	1	1
TRUE	Master Protein	A0A4X1UUF9	Nucleolar protein 10 OS=Sus scrofa OX=9823 GN=NOP10 PE=3 SV=1	20	1	1	1	64	7.6	9.99	2.83	1	1
TRUE	Master Protein	A0A480N9L6	Non-specific serine/threonine protein kinase OS=Sus scrofa OX=9823 PE=4 SV=1	5	1	1	1	1083	115.3	8.57	2.78	1	1
TRUE	Master Protein	A0A4X1V3M7	Small nuclear ribonucleoprotein E OS=Sus scrofa OX=9823 GN=SNRPE PE=3 SV=1	12	1	1	1	92	10.8	9.44	2.77	1	1
TRUE	Master Protein	A0A287A5P3	116 kDa U5 small nuclear ribonucleoprotein component OS=Sus scrofa OX=9823 GN=EF-TUD2 PE=1 SV=2	1	1	1	1	937	105.4	5.22	2.76	1	1
TRUE	Master Protein	A0A4X1VSZ6	SUN domain-containing protein OS=Sus scrofa OX=9823 GN=SUN2 PE=4 SV=1	2	1	1	1	713	79.7	7.02	2.75	1	1
TRUE	Master Protein	A0A480N4J2	Histone deacetylase OS=Sus scrofa OX=9823 PE=3 SV=1	3	1	1	1	359	40.7	7.46	2.74	1	1
TRUE	Master Protein	A0A4X1SJK8	40S ribosomal protein S24 OS=Sus scrofa OX=9823 GN=RPS24 PE=1 SV=1	11	1	1	1	132	15.3	10.92	2.73	1	1
TRUE	Master Protein	I3LI59	RNA-binding protein 8A OS=Sus scrofa OX=9823 GN=RBM8A PE=1 SV=2	6	1	1	1	174	19.9	5.72	2.71	1	1
TRUE	Master Protein	A0A480VPD1	26S proteasome regulatory subunit 6A (Fragment) OS=Sus scrofa OX=9823 PE=3 SV=1	4	1	1	1	446	50.1	5.29	2.7	1	1
TRUE	Master Protein	A0A4X1TM87	PKD domain-containing protein OS=Sus scrofa OX=9823 PE=3 SV=1	4	1	1	1	1077	119.6	7.28	2.7	1	1

TRUE	Master Protein	A0A4X1UU78	tRNA-binding domain-containing protein OS=Sus scrofa OX=9823 GN=AIMP1 PE=4 SV=1	4	1	1	1	335	37.5	6.8	2.7	1	1
TRUE	Master Protein	A0A4X1VUM6	Cold-inducible RNA-binding protein OS=Sus scrofa OX=9823 GN=DAZAP1 PE=4 SV=1	5	1	1	1	231	24.9	7.97	2.66	1	1
TRUE	Master Protein	I3LBT8	Small nuclear ribonucleoprotein-associated protein OS=Sus scrofa OX=9823 GN=SNRPN PE=3 SV=3	4	1	1	1	250	26.1	10.7	2.64	1	1
TRUE	Master Protein	A0A287ADF7	Prohibitin OS=Sus scrofa OX=9823 GN=PHB PE=1 SV=1	5	1	1	1	256	28.3	6.02	2.55	1	1
TRUE	Master Protein	A0A4X1TF03	C2 domain-containing protein OS=Sus scrofa OX=9823 PE=4 SV=1	3	1	1	1	684	73.4	6.7	2.55	1	1
TRUE	Master Protein	F2Z4Y8	40S ribosomal protein S11 OS=Sus scrofa OX=9823 GN=RPS11 PE=1 SV=1	7	1	1	1	158	18.4	10.3	2.54	1	1
TRUE	Master Protein	A0A4X1TUV5	U2 snRNP auxiliary factor large subunit OS=Sus scrofa OX=9823 GN=U2AF2 PE=3 SV=1	2	1	1	1	456	51.4	8.82	2.54	1	1
TRUE	Master Protein	A0A480JP88	E3 ubiquitin-protein ligase OS=Sus scrofa OX=9823 PE=4 SV=1	1	1	1	1	929	105.6	6.14	2.53	1	1
TRUE	Master Protein	A0A4X1VZZ5	S_100 domain-containing protein OS=Sus scrofa OX=9823 GN=S100A14 PE=4 SV=1	14	1	1	1	104	11.4	4.78	2.53	1	1
TRUE	Master Protein	A0A480Y0Q7	Cell cycle and apoptosis regulator protein 2 OS=Sus scrofa OX=9823 PE=4 SV=1	2	1	1	1	759	83.4	5.38	2.53	1	1
TRUE	Master Protein	A0A5S8KL32	Protein-serine/threonine phosphatase OS=Sus scrofa OX=9823 GN=PPP1CB PE=1 SV=1	5	1	1	1	303	34.4	6.4	2.52	1	1
TRUE	Master Protein	A0A4X1SET4	RRM domain-containing protein OS=Sus scrofa OX=9823 GN=NIFK PE=4 SV=1	4	1	1	1	294	34	10.15	2.52	1	1
TRUE	Master Protein	A0A4X1TVZ8	Nuclear cap-binding protein subunit 2 OS=Sus scrofa OX=9823 GN=NCBP2 PE=3 SV=1	7	1	1	1	156	18	8.21	2.51	1	1
TRUE	Master Protein	A0A286ZQJ3	SMP_C2CD2L domain-containing protein OS=Sus scrofa OX=9823 GN=C2CD2L PE=1 SV=1	1	1	1	1	669	71.7	8.32	2.51	1	1
TRUE	Master Protein	A0A5G2QY32	RPOLD domain-containing protein OS=Sus scrofa OX=9823 GN=POLR2C PE=1 SV=1	7	1	1	1	275	31.3	4.96	2.5	1	1
TRUE	Master Protein	A0A287A8V1	RNA helicase OS=Sus scrofa OX=9823 GN=EIF4A1 PE=1 SV=1	3	1	1	1	390	44.5	5.57	2.48	1	1
TRUE	Master Protein	A0A480U187	H/ACA ribonucleoprotein complex subunit 4 isoform 1 OS=Sus scrofa OX=9823 PE=3 SV=1	2	1	1	1	521	58.1	9.47	2.48	1	1
TRUE	Master Protein	A0A287APR1	60S ribosomal protein L18a OS=Sus scrofa OX=9823 GN=RPL18A PE=1 SV=1	6	1	1	1	224	26	10.92	2.48	1	1
TRUE	Master Protein	I3LNY6	IF rod domain-containing protein OS=Sus scrofa OX=9823 GN=NES PE=1 SV=2	1	1	1	1	1699	184.7	4.36	2.47	1	1
TRUE	Master Protein	A0A480TWW0	Signal transducer and activator of transcription OS=Sus scrofa OX=9823 PE=3 SV=1	1	1	1	1	787	89.9	6.11	2.46	1	1
TRUE	Master Protein	A0A4X1VLL6	BTB domain-containing protein OS=Sus scrofa OX=9823 PE=4 SV=1	2	1	1	1	490	53.6	9.16	2.46	1	1
TRUE	Master Protein	A0A5S6HGD8	Non-histone chromosomal protein HMG-17 OS=Sus scrofa OX=9823 GN=HMGN2 PE=3 SV=1	16	1	1	1	90	9.3	9.8	2.44	1	1

TRUE	Master Protein	A0A0U2VPN4	Estrogen related receptor gamma 1 OS=Sus scrofa OX=9823 GN=ESRRG1 PE=2 SV=1	5	1	1	1	458	51.2	6.48	2.44	1	1
TRUE	Master Protein	A0A287AWS4	60S ribosomal protein L27a OS=Sus scrofa OX=9823 GN=RPL27A PE=1 SV=2	7	1	1	1	148	16.6	11.12	2.43	1	1
TRUE	Master Protein	A0A480NBM2	Splicing factor 3B subunit 2 OS=Sus scrofa OX=9823 PE=4 SV=1	1	1	1	1	732	82.5	5.69	2.43	1	1
TRUE	Master Protein	A0A4X1TXJ8	60S ribosomal protein L30 OS=Sus scrofa OX=9823 GN=RPL30 PE=1 SV=1	10	1	1	1	115	12.8	9.63	2.42	1	1
TRUE	Master Protein	A0A0B8RSW3	Glyoxylate reductase 1 homolog OS=Sus scrofa domesticus OX=9825 GN=GLYR1 PE=3 SV=1	2	1	1	1	547	59.8	9.26	2.41	1	1
TRUE	Master Protein	A0A287ATC4	PRP6 homolog OS=Sus scrofa OX=9823 GN=PRPF6 PE=1 SV=1	1	1	1	1	1150	128.1	9.09	2.4	1	1
TRUE	Master Protein	A0A0B8RTA7	RNA binding motif protein 25 OS=Sus scrofa domesticus OX=9825 GN=RBM25 PE=4 SV=1	2	1	1	1	843	100.1	6.32	2.37	1	1
TRUE	Master Protein	A0A287AX73	KASH domain-containing protein OS=Sus scrofa OX=9823 GN=SYNE3 PE=1 SV=1	1	1	1	1	975	111.7	6.57	2.37	1	1
TRUE	Master Protein	A0A4X1THZ5	Ig-like domain-containing protein OS=Sus scrofa OX=9823 PE=4 SV=1	16	1	1	1	112	11.9	8.29	2.37	1	1
TRUE	Master Protein	A0A4X1UCP4	Homeobox protein NK-2 homolog E OS=Sus scrofa OX=9823 GN=NKX2-5 PE=3 SV=1	8	1	1	1	323	34.6	9.29	2.37	1	1
TRUE	Master Protein	A0A4X1VHE8	Glutamate dehydrogenase (NAD(P)(+)) OS=Sus scrofa OX=9823 GN=GLUD1 PE=3 SV=1	2	1	1	1	558	61.3	7.96	2.36	1	1
TRUE	Master Protein	A0A287AEM2	40S ribosomal protein S5 OS=Sus scrofa OX=9823 GN=RPS5 PE=1 SV=1	7	1	1	1	229	25.3	9.63	2.36	1	1
TRUE	Master Protein	A0A4X1SZJ9	Peptidylprolyl isomerase OS=Sus scrofa OX=9823 GN=FKBP5 PE=4 SV=1	2	1	1	1	465	52.2	5.69	2.36	1	1
TRUE	Master Protein	A0A287BJK8	MTTase N-terminal domain-containing protein OS=Sus scrofa OX=9823 GN=CDKAL1 PE=4 SV=1	5	1	1	1	550	61.7	8.31	2.35	1	1
TRUE	Master Protein	I3LP32	Pre-mRNA-splicing factor 38A OS=Sus scrofa OX=9823 GN=PRPF38A PE=3 SV=2	4	1	1	1	312	37.5	9.96	2.35	1	1
TRUE	Master Protein	A0A4X1VCR4	Creatine kinase OS=Sus scrofa OX=9823 GN=CKMT1A PE=3 SV=1	4	1	1	1	342	38.5	8.03	2.3	1	1
TRUE	Master Protein	A0A4X1U0Q7	RuvB-like helicase OS=Sus scrofa OX=9823 GN=RUVBL1 PE=3 SV=1	2	1	1	1	456	50.2	6.42	2.29	1	1
TRUE	Master Protein	Q9GMB0	Dolichyl-diphosphooligosaccharide--protein glycosyltransferase subunit 1 OS=Sus scrofa OX=9823 GN=RPN1 PE=1 SV=1	2	1	1	1	608	68.7	6.55	2.27	1	1
TRUE	Master Protein	F1S0S8	General transcription factor 3C polypeptide 4 OS=Sus scrofa OX=9823 GN=GTF3C4 PE=1 SV=2	1	1	1	1	679	76.7	7.36	2.26	1	1
TRUE	Master Protein	F1S4S0	Homeobox domain-containing protein OS=Sus scrofa OX=9823 GN=VAX1 PE=3 SV=1	3	1	1	1	333	34.6	9.38	2.22	1	1
TRUE	Master Protein	A0A480NQW0	CUGBP Elav-like family member 2 isoform 2 OS=Sus scrofa OX=9823 PE=3 SV=1	2	1	1	1	521	55.7	8.6	2.21	1	1
TRUE	Master Protein	A0A287A286	TRASH domain-containing protein OS=Sus scrofa OX=9823 GN=RPL24 PE=1 SV=1	4	1	1	1	217	24.7	11.5	2.21	1	1

TRUE	Master Protein	A0A287A961	Nipped-B protein OS=Sus scrofa OX=9823 GN=NIPBL PE=3 SV=2	0	1	1	1	2722	306.7	7.94	2.2	1	1
TRUE	Master Protein	A0A286ZW96	Homeobox domain-containing protein OS=Sus scrofa OX=9823 GN=SIX3 PE=4 SV=2	5	1	1	1	333	35.5	8.63	2.18	1	1
TRUE	Master Protein	A0A4X1SEZ3	Poly [ADP-ribose] polymerase OS=Sus scrofa OX=9823 PE=4 SV=1	3	1	1	1	502	56.2	8.78	2.18	1	1
TRUE	Master Protein	A0A287B8G0	Multifunctional fusion protein OS=Sus scrofa OX=9823 GN=PKM PE=1 SV=2	1	1	1	1	1090	121.2	7.96	2.18	1	1
TRUE	Master Protein	A0A4X1TZH0	Enhancer of rudimentary homolog OS=Sus scrofa OX=9823 PE=3 SV=1	7	1	1	1	176	20.2	6.2	2.17	1	1
TRUE	Master Protein	A0A5G2QT89	B-block_TFIIIC domain-containing protein OS=Sus scrofa OX=9823 GN=GTF3C1 PE=1 SV=1	0	1	1	1	2012	227.1	7.74	2.17	1	1
TRUE	Master Protein	A0A287AGI3	Splicing factor U2AF 35 kDa subunit OS=Sus scrofa OX=9823 GN=U2AF1 PE=4 SV=1	4	1	1	1	239	27.8	8.68	2.16	1	1
TRUE	Master Protein	I3LJ87	40S ribosomal protein S2 OS=Sus scrofa OX=9823 GN=RPS2 PE=1 SV=3	6	1	1	1	266	29.1	10.18	2.15	1	1
TRUE	Master Protein	I3LIR0	Polyhomeotic-like protein 2 isoform a OS=Sus scrofa OX=9823 GN=PHC2 PE=1 SV=2	1	1	1	1	887	93.7	9.03	2.15	1	1
TRUE	Master Protein	Q0QEM6	ATP synthase subunit beta (Fragment) OS=Sus scrofa OX=9823 GN=ATP5B PE=2 SV=1	4	1	1	1	437	47.1	5.11	2.14	1	1
TRUE	Master Protein	A0A481A1C4	Keratin, type I cytoskeletal 27 OS=Sus scrofa OX=9823 PE=4 SV=1	3	1	1	1	264	29.8	4.74	2.14	1	1
TRUE	Master Protein	A0A4X1TH57	Very-long-chain (3R)-3-hydroxyacyl-CoA dehydratase OS=Sus scrofa OX=9823 GN=HACD3 PE=3 SV=1	2	1	1	1	362	43.2	9.2	2.12	1	1
TRUE	Master Protein	A0A4X1SY17	Ribonucloprotein OS=Sus scrofa OX=9823 PE=3 SV=1	9	1	1	1	128	14.1	8.73	2.11	1	1
TRUE	Master Protein	A0A4X1VS56	Arf-GAP domain-containing protein OS=Sus scrofa OX=9823 GN=ARFGAP2 PE=4 SV=1	3	1	1	1	384	42	7.34	2.09	1	1
TRUE	Master Protein	G9F6X9	Dihydropyrimidinase-related protein 2 OS=Sus scrofa OX=9823 PE=2 SV=1	1	1	1	1	572	62.3	6.16	2.09	1	1
TRUE	Master Protein	A0A287BGL3	60S acidic ribosomal protein P0 OS=Sus scrofa OX=9823 GN=RPLP0 PE=3 SV=1	3	1	1	1	319	35.5	9.64	2.08	1	1
TRUE	Master Protein	F1S086	Calcium-binding mitochondrial carrier protein Aralar1 OS=Sus scrofa OX=9823 GN=SLC25A12 PE=1 SV=3	2	1	1	1	677	74.7	8.27	2.06	1	1
TRUE	Master Protein	A0A4X1UBG5	ATP-dependent RNA helicase DDX1 OS=Sus scrofa OX=9823 GN=DDX1 PE=3 SV=1	1	1	1	1	740	82.5	7.23	2.06	1	1
TRUE	Master Protein	A0A287BNP8	CRAL-TRIO domain-containing protein OS=Sus scrofa OX=9823 GN=RLBP1 PE=1 SV=1	2	1	1	1	319	37.1	5.33	2.06	1	1
TRUE	Master Protein	B6V8C8	40S ribosomal protein S3a OS=Sus scrofa OX=9823 GN=RPS3A PE=1 SV=1	3	1	1	1	264	29.9	9.74	2.05	1	1
TRUE	Master Protein	A0A4X1VU19	Robl_LC7 domain-containing protein OS=Sus scrofa OX=9823 GN=LAMTOR2 PE=3 SV=1	8	1	1	1	125	13.5	5.4	2.04	1	1
TRUE	Master Protein	A0A4X1WCH9	SWIB domain-containing protein OS=Sus scrofa OX=9823 GN=SMARCD1 PE=3 SV=1	3	1	1	1	515	58.2	9.25	2.04	1	1

TRUE	Master Protein	A0A5G2QWT4	Alpha_adaptinC2 domain-containing protein OS=Sus scrofa OX=9823 GN=AP2A2 PE=1 SV=1	1	1	1	1	857	94.2	6.27	2.03	1	1
TRUE	Master Protein	A0A4X1SW39	Sodium/potassium-transporting ATPase subunit alpha OS=Sus scrofa OX=9823 GN=ATP1A1 PE=3 SV=1	1	1	1	1	1018	112.3	5.68	2.03	1	1
TRUE	Master Protein	A0A287A4H2	Transcriptional activator protein Pur-beta OS=Sus scrofa OX=9823 GN=PURB PE=1 SV=1	2	1	1	1	308	32.9	5.43	2.03	1	1
TRUE	Master Protein	A0A287AZ57	Nsp1_C domain-containing protein OS=Sus scrofa OX=9823 GN=NUP62 PE=1 SV=1	2	1	1	1	478	49.1	5.43	2.02	1	1
TRUE	Master Protein	A0A286ZPW3	WD_REPEATS_REGION domain-containing protein OS=Sus scrofa OX=9823 GN=STRN3 PE=1 SV=1	1	1	1	1	760	83	5.17	2.02	1	1
TRUE	Master Protein	A0A4X1SYE0	Carbonate dehydratase IV OS=Sus scrofa OX=9823 PE=3 SV=1	4	1	1	1	311	35.2	7.53	2.02	1	1
TRUE	Master Protein	F1SGD0	F-box-like/WD repeat-containing protein TBL1XR1 isoform 1 OS=Sus scrofa OX=9823 GN=TBL1XR1 PE=4 SV=3	3	1	1	1	514	55.6	5.55	2	1	1
TRUE	Master Protein	A0A287A452	Ribosomal protein L15 OS=Sus scrofa OX=9823 GN=RPL15 PE=1 SV=1	4	1	1	1	187	21.9	10.92	2	1	1
TRUE	Master Protein	A0A287BIZ5	Protein RUFY3 isoform X4 OS=Sus scrofa OX=9823 GN=RUFY3 PE=1 SV=1	2	1	1	1	597	67.8	5.87	2	1	1
TRUE	Master Protein	A0A4X1UKP8	DNA topoisomerase 2 OS=Sus scrofa OX=9823 GN=TOP2B PE=3 SV=1	1	1	1	1	1539	173.9	8.02	2	1	1
TRUE	Master Protein	A0A4X1UCV9	Importin N-terminal domain-containing protein OS=Sus scrofa OX=9823 GN=TNPO1 PE=4 SV=1	1	1	1	1	844	95.7	4.98	1.99	1	1
TRUE	Master Protein	A0A4X1TQ26	SCD domain-containing protein OS=Sus scrofa OX=9823 GN=STAG1 PE=3 SV=1	1	1	1	1	1227	140.9	5.57	1.99	1	1
TRUE	Master Protein	A0A287B109	UPAR/Ly6 domain-containing protein OS=Sus scrofa OX=9823 GN=LYNX1 PE=4 SV=1	9	1	1	1	116	12.5	7.05	1.99	1	1
TRUE	Master Protein	K9J6I7	Adducin 1 (Alpha) OS=Sus scrofa OX=9823 GN=ADD1 PE=2 SV=1	1	1	1	1	744	81.2	5.48	1.98	1	1
TRUE	Master Protein	A0A4X1STI8	Non-specific serine/threonine protein kinase OS=Sus scrofa OX=9823 GN=RPS6KA5 PE=3 SV=1	1	1	1	1	957	105.6	7.96	1.98	1	1
TRUE	Master Protein	A0A480JG04	Splicing factor 3B subunit 3 OS=Sus scrofa OX=9823 PE=4 SV=1	1	1	1	1	1118	124.2	5.31	1.98	1	1
TRUE	Master Protein	A0A4X1UUJ1	Kinesin-like protein OS=Sus scrofa OX=9823 PE=3 SV=1	2	1	1	1	679	76.9	6.4	1.98	1	1
TRUE	Master Protein	A0A2C9F391	Major prion protein OS=Sus scrofa OX=9823 GN=PRNP PE=1 SV=1	3	1	1	1	256	27.6	9.32	1.97	1	1
TRUE	Master Protein	A0A4X1TXT9	E3 ubiquitin-protein ligase TRIP12 OS=Sus scrofa OX=9823 GN=TRIP12 PE=3 SV=1	1	1	1	1	1983	219.1	8.29	1.97	1	1
TRUE	Master Protein	F1RPW9	Elongation factor 1-gamma OS=Sus scrofa OX=9823 GN=EEF1G PE=4 SV=3	3	1	1	1	436	49.9	6.57	1.97	1	1
TRUE	Master Protein	A0PFK6	F-actin-capping protein subunit alpha OS=Sus scrofa OX=9823 GN=CP alpha2 PE=2 SV=1	5	1	1	1	286	33	5.85	1.97	1	1
TRUE	Master Protein	A0A480QJH2	BCL2-associated athanogene 6 OS=Sus scrofa OX=9823 PE=4 SV=1	2	1	1	1	522	55.1	5.69	1.97	1	1

TRUE	Master Protein	A0A2I2KHU2	Genome polyprotein (Fragment) OS=Atypical porcine pestivirus OX=1914447 GN=NS2-3 PE=4 SV=1	2	1	1	1	962	107.6	7.12	1.96	1	1
TRUE	Master Protein	A0A480JRB0	Clathrin light chain OS=Sus scrofa OX=9823 PE=3 SV=1	3	1	1	1	229	25.2	4.6	1.95	1	1
TRUE	Master Protein	A0A5G2QWH1	V-type proton ATPase subunit H OS=Sus scrofa OX=9823 GN=ATP6V1H PE=3 SV=1	4	1	1	1	464	53.7	6.61	1.95	1	1
TRUE	Master Protein	F1S8K5	FACT complex subunit OS=Sus scrofa OX=9823 GN=SUPT16H PE=1 SV=2	1	1	1	1	1083	123.6	5.78	1.95	1	1
TRUE	Master Protein	P62831	60S ribosomal protein L23 OS=Sus scrofa OX=9823 GN=RPL23 PE=1 SV=1	6	1	1	1	140	14.9	10.51	1.94	1	1
TRUE	Master Protein	D2XZ48	Non-structural protein 6 OS=Porcine reproductive and respiratory syndrome virus OX=28344 PE=2 SV=1	0	1	1	1	2502	271.2	7.5	1.93	1	1
TRUE	Master Protein	A0A287A1N9	Zinc finger protein 385A isoform a OS=Sus scrofa OX=9823 GN=ZNF385A PE=1 SV=2	3	1	1	1	387	40.6	9.86	1.93	1	1
TRUE	Master Protein	A0A481D3P3	Transmembrane protein 43 OS=Sus scrofa OX=9823 PE=3 SV=1	2	1	1	1	400	44.9	7.71	1.92	1	1
TRUE	Master Protein	A0A480MRU2	DNA repair protein RAD50 OS=Sus scrofa OX=9823 PE=4 SV=1	2	1	1	1	511	59.5	8.73	1.92	1	1
TRUE	Master Protein	A0A287A3R3	PDZ domain-containing protein OS=Sus scrofa OX=9823 GN=GIPC3 PE=3 SV=2	3	1	1	1	314	34.3	5.6	1.92	1	1
TRUE	Master Protein	F1RNL4	Serine/threonine-protein phosphatase OS=Sus scrofa OX=9823 GN=PPP1CC PE=1 SV=4	3	1	1	1	304	34.9	5.58	1.9	1	1
TRUE	Master Protein	K7GNY4	60S ribosomal protein L8 OS=Sus scrofa OX=9823 GN=RPL8 PE=1 SV=3	5	1	1	1	225	24.6	11.02	1.9	1	1
TRUE	Master Protein	A0A480J6I6	Lysine-specific demethylase 4A isoform X1 OS=Sus scrofa OX=9823 PE=4 SV=1	1	1	1	1	821	91.8	5.33	1.9	1	1
TRUE	Master Protein	A0A286ZJL5	V-type proton ATPase subunit D OS=Sus scrofa OX=9823 GN=ATP6V1D PE=1 SV=1	3	1	1	1	247	28.2	9.36	1.9	1	1
TRUE	Master Protein	K9IWE3	Histone-lysine N-methyltransferase ASH1L OS=Sus scrofa OX=9823 GN=ASH1L PE=2 SV=2	0	1	1	1	2958	331.4	9.38	1.9	1	1
								220819	25034.2	3396285.408	10606.09	8317.3	386

Table S3. Matrisome compoenents of retinal decellularized extracellular matrix (RdECM).

Division	Category	Name
Core Matrisome	Collagen	Collagen, type XVIII, alpha 1
	Proteoglycan	Heparan sulfate proteoglycan 2
	ECM Glycoprotein	Agrin
		Laminin, beta 2 (laminin S)
		Laminin, gamma 1 (formerly LAMB2)
		Nidogen 2 (osteonidogen)
		Tubulointerstitial nephritis antigen-like 1
		Tenascin R (restrictin, janusin)
		Von Willebrand factor A domain containing 1
Matrisome-associated	Secreted factor	Fibroblast growth factor 2 (basic)
		S100 calcium binding protein A14
	ECM-affiliated protein	Lectin, galactoside-binding, soluble, 1
